# Modern approaches and future prospects in lettuce breeding for controlled environment agriculture

**DOI:** 10.3389/fpls.2026.1870041

**Published:** 2026-09-03

**Authors:** Ajaya Poudel, Asmita Bhusal, Krishna Bhattarai

**Affiliations:** 1 Texas A&M AgriLife Research and Extension Center, Department of Horticultural Sciences, Texas A&M University, Dallas, TX, United States; 2 Department of Horticultural Sciences, Texas A&M University, College Station, TX, United States

**Keywords:** host resistance, *Lactuca sativa*, omics-informed breeding, resource use efficiency, trait optimization

## Abstract

Controlled environment agriculture (CEA) is expanding rapidly as a climate-resilient approach for year-round lettuce production near consumption centers. However, despite its supply chain advantages and high productivity, the economic viability and scalability of CEA-grown lettuce remain constrained by the continued reliance on cultivars developed for variable open-field conditions rather than stable, high-input, and space-limited environments of indoor production systems. This mismatch between existing germplasm and CEA-specific production requirements represents a critical gap that has not been comprehensively addressed in lettuce breeding. This review discusses the need for a paradigm shift in breeding priorities toward cultivars specifically optimized for CEA, with an emphasis on traits that improve output per unit of energy, time, and growing area. Key breeding targets include accelerated growth and shortened crop cycles, enhanced light-use efficiency and photosynthetic performance, improved tolerance to temperature extremes, compact plant architecture for high-density production, optimized shoot-to-root biomass partitioning for hydroponic systems, and resilience to major CEA-associated physiological disorders, pests, and diseases. Because CEA reduces many field-related stressors, breeding efforts can place greater emphasis on consumer- and market-oriented traits, including consistent product quality, enhanced nutritional value, and uniformity compatible with automated production systems. This review consolidates dispersed evidence on these CEA-targeted traits and links them to a modern lettuce breeding toolbox, providing a framework to guide cultivar development for controlled environments. We outline how integrating approaches ranging from conventional genetics and marker-assisted selection to genomic selection and gene-editing technologies such as CRISPR can accelerate genetic gain for these complex trait combinations. Furthermore, the integration of high-throughput phenotyping, artificial intelligence, and multi-omics platforms offers a powerful, data-driven approach to decipher genotype-by-environment interactions and optimize lettuce for controlled environments. By identifying critical knowledge gaps and outlining a targeted breeding framework, this review provides a roadmap for developing resource-efficient, high-performing, quality-focused lettuce cultivars tailored to next-generation CEA, thereby improving the industry’s profitability, sustainability and ability to meet the food demands of a rapidly urbanizing global population.

## 1 Introduction

Lettuce (*Lactuca sativa* L.), a cool season diploid (2n = 2x = 18) leafy green, is one of the most widely consumed and economically significant vegetable crops worldwide, serving as a key component of the fresh-cut and ready-to-eat produce market. Global lettuce production reached 28.2 million tons in 2024 FAOSTAT (2025), led by China, while the United States (U.S.) produced 5.1 million tons in 2025, representing a 24.97% increase compared to a decade earlier. This production, valued at $5.8 billion across head, leaf, and romaine types, represented approximately 30.72% of the nation’s total vegetable production value (USDA-NASS, 2026). Lettuce also dominates the organic vegetable sector, with a market value of approximately $275.6 million, accounting for 14% of total organic sales and 19% of harvested acreage in 2021 (Davis et al., 2024; USDA-NASS, 2022). By 2023, lettuce, excluding head lettuce, was the highest-exported fresh organic vegetable, with an export value of $62.3 million (Davis et al., 2024; Raszap Skorbiansky, 2025). In the U.S., lettuce is the most consumed leafy green vegetable, with a per capita availability of 41 pounds of head, romaine, and iceberg lettuce (Davis et al., 2025). As salad consumption continues to rise, lettuce remains a crucial source of dietary nutrients and health-promoting phytochemicals and bioactive compounds, including flavonoids, antioxidants, phenolics, chlorophyll, carotenoids, Vitamin B, C, and E, and sesquiterpene lactones (Shi et al., 2022; Yang et al., 2022; Kim et al., 2016).

Although most lettuce in the U.S. is field grown in California and Arizona, this geographic concentration increases vulnerability to climate-driven disruptions. For instance, severe spring weather delayed planting, and flooding caused an estimated $324.1 million in crop losses in 2023 in California’s Salinas valley, a region responsible for approximately 60% of the U.S. lettuce production (Lee, 2023). Because lettuce is sensitive to elevated temperatures, rising temperatures can inhibit germination and shorten growth periods, thereby reducing marketability and yield due to physiological disorders such as bolting and tipburn (Thompson et al., 1979; Jenni and Hayes, 2010; Lafta et al., 2021; Kreutz et al., 2021). Moreover, long transportation distances, or “food miles” from concentrated production regions increase distribution costs, carbon emissions, and postharvest losses (Pennisi et al., 2025; Maynard et al., 2023; Martin et al., 2023). As the impact of climate change intensifies, increasing the frequency and severity of extreme weather events over the past decades, traditional field production faces growing uncertainty (Bolster et al., 2023). These challenges, coupled with rising labor and input expenses, have accelerated interest in production systems that offer higher efficiency and a more stable supply. Rising consumer demand for year-round, locally produced leafy greens has driven significant expansion of CEA. Between 2014 and 2019, the total area of CEA-grown lettuce in the U.S. increased by 28%, while production more than doubled to about 25 million kg across 0.5 million square meters (Dohlman et al., 2024). CEA is rapidly gaining global attention for its ability to enhance resource-use efficiency (RUE), crop quality, and productivity by precisely regulating key environmental factors such as temperature, humidity, light, nutrients, and carbon dioxide (CO_2_) (Calvo-Baltanás et al., 2025; Shamshiri et al., 2018; Gómez et al., 2019). CEA systems range from simple plastic tunnels to advanced greenhouses and vertical farms and are increasingly integrated into urban agriculture, including home gardens, rooftop greenhouses, and large-scale warehouse-based plant factories (Jansen et al., 2016; Eaves and Eaves, 2018; Mitchell, 2022). By enabling consistent, year-round production close to consumption centers, CEA reduces transportation distances, minimizes postharvest losses, and lowers emissions and distribution costs (Gómez et al., 2019). Furthermore, the adoption of CEA by states with minimal open-field production has diversified the domestic supply chain. As climate change, pest pressure, and water scarcity continue to challenge conventional systems, CEA offers a sustainable and resilient alternative capable of meeting future demands for fresh high-quality lettuce. Average lettuce yield has been reported to be higher in CEA (3.68 kg/m_-2_), especially in vertical farms (an average of 6.88 kg/m^2^) than in field production (1.88 kg/m^2^) largely because of shortened crop cycles averaging 40 days (with the vast majority of CEA trials completed in 70 days or less) compared to 60–120 days in field, enabling multiple production cycles per year (Gargaro et al., 2023; Bhattarai et al., 2025).

Despite the rapid expansion of CEA, the economic viability and scalability of CEA-grown lettuce remain constrained by a fundamental and largely unresolved challenge: most commercial lettuce cultivars currently used in indoor production systems were developed for the variable and often unpredictable conditions of open-field agriculture rather than the highly controlled, resource-intensive, and space-limited environments characteristic of CEA (Hayes, 2018; Bhattarai et al., 2025). Consequently, many existing cultivars may not perform optimally under CEA-specific conditions or fully exploit the unique opportunities for enhanced productivity, RUE, and quality that these systems offer. Several reviews have addressed important aspects of lettuce improvement, including genetics and molecular and conventional breeding strategies (Salem et al., 2023; Hassan et al., 2021; Sandoya, 2019; Hayes, 2018), disease resistance and marker-assisted selection (Simko, 2013), environmental management (Boros et al., 2023; Ahmed et al., 2020), and postharvest longevity (Peng and Simko, 2023). More recently, Bhattarai et al. (2025) briefly summarized breeding targets and available breeding tools for lettuce and other horticultural crops in CEA systems. However, a comprehensive synthesis of the biological, economic, and operational rationale that can serve as a guideline for breeding lettuce for CEA is needed. This review highlights a full range of CEA-targeted breeding priorities, or integrated conventional and emerging breeding approaches into a unified framework for lettuce cultivar development in indoor production systems. Specifically, it covers: (i) the rationale for shifting breeding priorities away from broad field adaptation toward CEA-optimized performance; (ii) key breeding targets, including accelerated growth, improved RUE, compact plant architecture, resistance to CEA-specific biotic and abiotic stressors, and enhanced nutritional and postharvest quality; and (iii) the breeding strategies and tools available to achieve these objectives, including conventional breeding, marker-assisted selection, genomic selection, multi-omics approaches, AI-enabled high-throughput phenotyping, and genome editing technologies. The scope of this review is limited to lettuce grown in CEA systems, including hydroponics, greenhouses, high tunnels, vertical farms, and plant factories with artificial lighting. Open-field production and postharvest technologies are discussed only where directly relevant to breeding objectives. By explicitly linking breeding goals with the economic and operational realities of CEA, this review provides a framework to guide the development of resource-efficient, high-quality, and highly productive lettuce cultivars that can enhance the profitability, sustainability, and resilience of the rapidly growing CEA industry.

## 2 Significance of breeding lettuce for CEA

Crop productivity is determined by the interaction between genetic potential and environmental conditions. Accordingly, traditional lettuce breeding has largely emphasized stable performance across heterogeneous field conditions, where cultivars must maintain consistent yield and quality despite variation in temperature, moisture, and pest and disease pressure. In contrast, CEA minimizes much of the environmental variability through precise regulation of temperature, humidity, light, nutrients, and CO_2_, allowing genotypes to express their genetic potential in a more predictable and uniform manner (Bhattarai et al., 2025; Folta, 2019). This shift from variable to highly regulated environments creates an important opportunity to redefine breeding goals. Rather than prioritizing broad field adaptation, breeders can target performance under the defined and repeatable conditions of commercial indoor and protected systems (Kim and van Iersel, 2022).

Historically, lettuce improvement has largely targeted performance and adaptation to open-field environments (Hayes, 2018). As a result, most cultivars currently used in hydroponic and indoor production were originally developed for outdoor soil-based cultivation, where the primary biological and economic challenges differ substantially from those in CEA. In indoor systems, profitability is constrained less by land availability than by high establishment and operating costs and limited growing space, especially in plant factories with artificial lighting (PFALs) and vertical farms. Energy use is a central component of this cost structure. Lighting and temperature regulation account for a large proportion of operating expenses, with lighting alone representing approximately 10–50% of total operational expenses in some systems (van Iersel, 2017; Kozai, 2018; Watson et al., 2018). A European Commission analysis reported that indoor vertical farms spend up to approximately 60% of their revenue on electricity alone, while only 27% of such businesses were profitable (European Union, 2023). Recent industry experience further underscores these economic constraints. Several prominent vertical farming companies have faced insolvency, bankruptcy, or major downsizing, in part because capital-intensive infrastructure and high operating costs, particularly for energy, have proven difficult to offset when production is concentrated on relatively low-margin crops such as leafy greens (Chandra and Lal, 2026; Phua, 2023). Although these failures cannot be attributable to a single cause, they collectively indicate that structural improvements alone, including more efficient LEDs, improved HVAC systems, and automation, have not yet been sufficient to close the economic gap. This consideration leaves one important aspect of the system, namely, cultivar improvement and breeding. If CEA is to become more broadly viable, profitability will depend not only on advances in facility design and operational efficiency, but also on the biological optimization of the crop itself. Cultivars that generate more marketable biomass per unit of energy, nutrients, water, space, and time can directly improve production efficiency and unit economics. In this respect, genetic improvement offers a particularly attractive complement to engineering innovation because, once desirable alleles are incorporated into elite cultivars, their benefits can be deployed repeatedly without the recurring capital expenditures associated with infrastructure upgrades.

These economic realities reshape breeding priorities for lettuce in CEA. Traits that improve output per unit of time and resource invested, especially growth rate and RUE, become central breeding targets. Similarly, traits that enhance compatibility with indoor production technologies, including improved light-use efficiency (LUE), uniformity under controlled conditions, and suitability for mechanized harvesting, gain importance alongside nutritional quality and market appeal. This represents a shift from historical field-based breeding, which has often prioritized traits such as head weight and crop stature in different lettuce types. However, in CEA, romaine, butterhead, and leaf lettuce, including their subtypes, are the most widely cultivated forms of lettuce.

A core rationale for breeding specifically for CEA is that genetic improvements can contribute to lowering production costs and improving the sustainability of the production system. Enhanced LUE is especially important in PFAL systems, where production depends entirely on artificial lighting and the conversion of electrical energy into photosynthetically active radiation and then into plant biomass (Kozai et al., 2019). Consequently, genotypes with greater photosynthetic capacity or the ability to maintain marketable yields under lower light intensity could reduce electricity demand without compromising productivity (Folta, 2019). Similarly, improved tolerance to heat or cold stress could reduce the need for intensive heating and cooling, thereby lowering climate-control costs and improving overall system efficiency and resilience. Genetic gains in growth rate are equally important because CEA systems can support far more crop cycles per year than field production systems, with 11–12 cycles per year in CEA compared with 1–2 in the open field (USEPA, 2007). Even modest reductions in time to harvest can therefore have significant effects on annual productivity and return on investment. In addition, the environmental controllability of CEA facilities allows them to function as speed breeding platforms, where generation time and selection cycles can be accelerated through the manipulation of photoperiod and temperature (Sharma et al., 2024; Chiurugwi et al., 2019; Xu et al., 2023). At the same time, these gains must be interpreted realistically. Breeding can improve biological efficiency, but it cannot by itself eliminate the underlying capital and energy burdens of indoor farming. The long-term viability of CEA will therefore depend on the combined effects of genetic improvement, engineering innovation, and economically sound production models.

Prioritization of plant architecture and crop uniformity traits provides a clear example of how breeding objectives shift under CEA. In multi-tier systems, where vertical space is limited, compact canopy architecture becomes far more valuable than in the open field or even in conventional single-tiered greenhouse production systems. Uniformity in plant height, canopy form, and growth rate facilitates high-density planting, reduces interference among tiers, and improves compatibility with mechanized harvesting and automation, which are increasingly important under labor scarcity and high labor costs in indoor production (Bhattarai et al., 2025). Recent advances in the genetic dissection of architecture-related traits, including genes affecting leaf angle, curvature, and compactness, offer actionable targets for developing lettuce ideotypes suited to vertical farming systems (Chen et al., 2025; Xie et al., 2024). It is worth noting that breeding for automation compatibility addresses an economic bottleneck that cannot be solved by facility design and technological innovations alone. Robotic systems perform best when crop structure is predictable and uniform, underscoring the close interdependence between cultivar genetics and production-system engineering.

Beyond efficiency, breeding for CEA has strategic importance for product differentiation and market value. Breeders can place greater emphasis on quality traits that are often difficult to maintain in field conditions, including nutritional composition, visual appeal, flavor, texture, and postharvest performance (Bhattarai et al., 2025; Kreuger et al., 2018). For example, red leaf cultivars often exhibit weaker or less stable anthocyanin accumulation under artificial lighting (Wada et al., 2022), highlighting a specific need to breed cultivars with reliable pigmentation under indoor lighting regimes. Postharvest traits are equally important for fresh produce markets, where enzymatic discoloration and tissue deterioration are major determinants of shelf life and consumer acceptance. The relatively high heritability of these traits makes them amenable to improvement through phenotyping and genetic selection (Peng and Simko, 2023). However, recent CEA failures also demonstrate that premium quality alone is unlikely to sustain profitability if production costs remain too high relative to field-grown alternatives. For that reason, the most effective breeding strategies for CEA will likely be those that integrate quality enhancement with improvements in biological and economic efficiency, rather than treating these objectives separately. Taken together, these considerations highlight why breeding lettuce specifically for CEA is both biologically justified and economically necessary. CEA imposes a distinct set of constraints and opportunities, including pressure to maximize energy and time efficiency, optimize performance in space-limited production systems, manage protected-system diseases and disorders, and deliver a consistent, high-value product. Cultivars optimized for these conditions will be essential for improving profitability, sustainability, and the long-term scalability of CEA lettuce production. Importantly, genetic improvement and engineering innovation should not be viewed as competing strategies but as complementary components of the same production framework. Advances in lighting efficiency, climate control, and automation can reduce system costs, but the extent to which those gains are converted into marketable yield, quality, and profitability also depends on the genetic capacity of the cultivar being grown.

## 3 Breeding priorities for CEA lettuce

**Figure 1:**
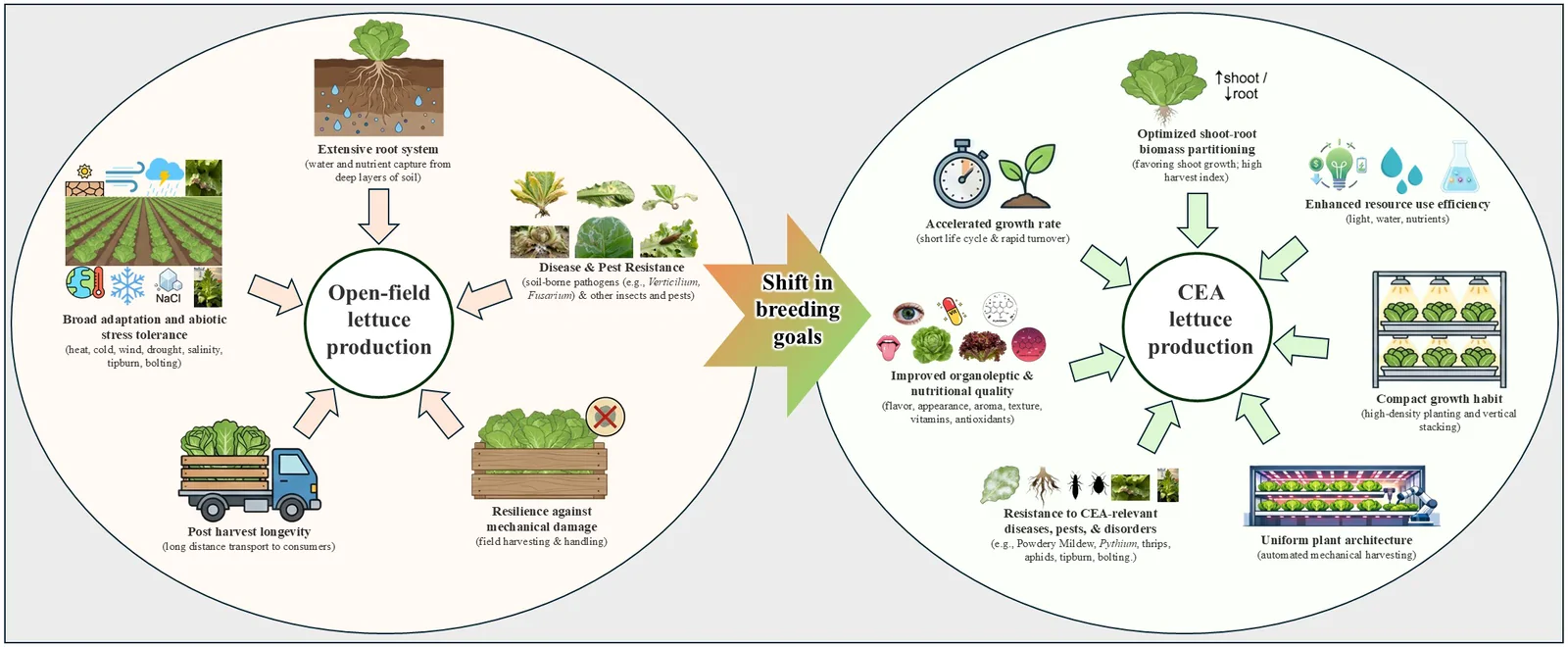
A comparative representation of breeding goals for lettuce targeted to open-field and controlled environment agriculture production systems.

In addition to traditional goals such as yield, nutritional quality, and resistance to pests and diseases, the growing adoption of CEA necessitates the development of lettuce cultivars specifically adapted to indoor production systems. Unlike conventional open-field production, CEA environments impose unique and optimized growing conditions. As a result, breeding priorities for lettuce in CEA must extend beyond conventional targets to include traits that maximize performance, uniformity, and economic return under these specialized conditions. Comparative breeding priorities for CEA in relation to open-field production are shown in **Figure 1**.

### 3.1 Enhanced growth rate and yield potential

In CEA, profitability is associated with the amount of marketable biomass produced per unit area, time, and energy invested (Calvo-Baltanás et al., 2025; Kaiser et al., 2024). Faster growth rates translate into rapid crop turnover, which is critical for lowering overall production costs, primarily energy cost for lighting and climate control per unit of crop produced (Teo and Yu, 2024; Folta, 2019). Because lighting and climate control dominate operating expenses, faster biomass accumulation and shorter crop cycles reduce energy costs by increasing turnover and distributing fixed costs across harvests. Consequently, rapid vegetative growth is a primary breeding objective. Moreover, compared with open-field production, CEA systems can support substantially more production cycles, so even modest reductions in time to harvest can generate large annual productivity gains at the facility level. Hydroponic production can shorten crop cycles compared with soil systems, as demonstrated in a study in which lettuce matured in 40.5 days under deep-water culture (DWC) whereas it took 48 days in soil, reducing the total growing cycle from 107 to 92.5 days (Majid et al., 2021; Dohlman et al., 2024; Niu and Masabni, 2022). However, faster shoot growth in CEA lettuce is not physiologically unconstrained. Tipburn arises when calcium demand in rapidly expanding inner leaves exceeds calcium supply, and localized Ca^2+^ deficiency can occur even when there is sufficient Ca^2+^ in the root-zone, if transpiration is limited by high relative humidity and inadequate air movement, or when growth-promoting conditions such as high light and temperature accelerate leaf expansion and increase Ca^2+^ demand beyond the plant’s delivery capacity (Saure, 1998; Barta and Tibbitts, 2000; Frantz et al., 2004; Moosavi-Nezhad and Meng, 2025). As a result, management strategies that slow growth, including reducing light intensity or temperature, or harvesting earlier before full head enclosure, can reduce tipburn but also compromise yield, underscoring a fundamental trade-off between maximizing growth rate and maintaining physiological integrity in rapidly growing canopies (Collier and Tibbitts, 1984; Ertle and Kubota, 2023b; Moosavi-Nezhad and Meng, 2025).

Traits that contribute to high biomass accumulation and rapid growth rate, are essential for maximizing output in the limited space of vertical farming, especially for leafy greens like lettuce, where vegetative growth constitutes the harvested product. Therefore, maximizing edible shoot biomass is a central goal in lettuce breeding, regardless of the production system. In open-field agriculture, a robust and extensive root system is essential for acquiring water and nutrients from the deeper soil layers. For instance, in field crops like rice, deep root systems are vital for accessing water and nutrients under drought conditions, with genes such as *OsPTR9* enhancing nutrient uptake and contributing to yield stability (Zhao et al., 2021). In hydroponics, water and nutrients are continuously delivered to roots, making large root systems less advantageous and potentially inefficient. Redirecting energy from excess root growth to shoots can improve harvest index (HI) and yield. Consequently, a major objective in CEA lettuce breeding is to optimize the shoot-to-root biomass ratio by selecting genotypes with smaller yet more efficient root systems. Plant-level modelling for vertical farms further shows that current cultivars approach sink-limited yields as photosynthetic photon flux density (PPFD) increases, with no additional yield benefit beyond ~287 μmol m^-2^ s^-1^ at 22 °C despite higher light availability (Meeuws et al., 2025). In “NextGen” scenarios with faster intrinsic growth and higher plant densities, lettuce yields become strongly sink-limited even when light interception and photoperiod are optimized, indicating that ultra-fast growth is ultimately bound by sink strength and tissue capacity to store and utilize assimilates rather than by light supply alone (Meeuws et al., 2025). Together, these findings imply that breeding for accelerated growth in CEA lettuce cannot focus solely on increasing photosynthetic capacity or shortening crop cycles. At the canopy level, CEA lettuce increasingly encounters sink-limited conditions as LUE and PPFD are optimized, prompting internal regulatory responses such as reduced photosynthetic efficiency, constrained leaf expansion, or greater allocation of assimilates to non-harvestable tissues, which lower the marginal return of additional inputs (Meeuws et al., 2025). At the organ level, rapid leaf expansion under high light and temperature can outpace Ca^2+^ delivery to enclosed young leaves, causing tipburn even when root-zone Ca^2+^ is adequate, so ultra-fast growth aggravates a source–sink imbalance for calcium as well as carbon (Moosavi-Nezhad and Meng, 2025). Breeding ideotypes for CEA must therefore combine higher sink capacity and stable carbon partitioning (e.g., improved harvest index, controlled leaf expansion) with efficient Ca^2+^ translocation and genetic tipburn resistance, enabling high turnover while maintaining canopy integrity and marketable quality under sink-limited, high-input conditions.

### 3.2 Resource use efficiency

#### 3.2.1 Light use efficiency and photosynthetic performance under electric lighting

Light, the most precisely controllable factor, plays a critical role in optimizing growth. In contrast to greenhouses that utilize a hybrid lighting system combining natural sunlight and supplemental electric lighting, indoor vertical farms depend exclusively on artificial illumination. In a plant factory with artificial lighting (PFAL), electrical energy is first transformed into light, which is subsequently converted by plants into chemical energy through photosynthesis (Kozai et al., 2019). Given that lighting expenses can represent 10–50% of total operating costs in CEA (van Iersel, 2017; Kozai, 2018; Watson et al., 2018), they play a critical role in determining overall economic viability. In CEA, breeding must shift from maximizing biomass to optimizing the conversion of energy into marketable yield. This makes LUE, defined as biomass produced per unit of incident light (g/mol of incident light), a key trait, with high-LUE lettuce varieties essential for improving the profitability and sustainability of CEA operations.

Future lettuce cultivars for CEA may be bred to perform under low light (<100 μmol m^-2^ s^-1^, ~1/20th of full sunlight, or ~6 mol·m^-2^·day^-1^ daily light integral), reducing photon requirements while maintaining marketable quality (Folta, 2019). Genetics and breeding approaches can identify and target LUE related traits, such as chlorophyll content, ΦPSII (quantum yield of Photosystem II), and the electron transport rate using tools such as CRISPR/Cas9 (Bhattarai et al., 2025; Jayalath, 2023). For example, editing the Phytochrome Interacting Factor 4 (*PIF4*) gene improved light response and plant development in tomato (Kunta et al., 2025). Importantly, however, the response to LED spectra is genotype-dependent because cultivars differ in how they translate spectral composition into biomass accumulation, photosynthetic performance, and nutritional quality, and this genotype × LED spectrum interaction represents an underexplored but commercially critical dimension of CEA lettuce breeding. In hydroponic ‘Italian Bolting-Resistant’ lettuce grown at constant PPFD, different red:blue (R:B) ratios produced distinct trait-specific optima R:B 60:40 enhanced root development, chlorophyll accumulation, antioxidant capacity, and nitrate reduction, whereas R:B 80:20 supported robust biomass production together with balanced N, Ca, and Mg accumulation, leading to cultivar-specific spectral recommendations rather than a single ideal spectrum (Hu et al., 2026). In Batavia lettuce, a different optimum was found, as R:B 70:30 produced the highest chlorophyll a, chlorophyll b, carotenoids, and leaf relative water content compared with R:B 80:20 and 90:10, further illustrating that spectral requirements vary among lettuce types (Karimi et al., 2026).

Spectral responses also involve clear physiological trade-offs that are directly relevant to breeding. In red lettuce, increasing the blue fraction from 12.5% to 40% under an R:B background decreased fresh weight, soluble sugars, and starch, but increased chlorophyll, carotenoids, anthocyanins, flavonoids, phenolic compounds, and most mineral elements; by contrast, supplemental far-red increased fresh and dry mass, leaf area, soluble sugars, and starch, but reduced anthocyanin concentration and several minerals (Van Brenk et al., 2024). These results indicate that blue-enriched spectra often favor pigment accumulation and nutritional quality, whereas far-red can enhance canopy expansion and biomass, meaning the optimal light recipe depends on whether breeding targets prioritize yield, coloration, nutritional density, or a balance among them.

Mechanistically, different LED spectra influence how photosynthetic energy is processed. Physiological analyses of lettuce under contrasting red and blue spectra showed that red-dominant light favored photosynthetic activity and growth, whereas blue-dominant light induced stress-associated responses, including activation of cyclic electron flow around Photosystem I and increased respiration, which may contribute to slower growth despite potential gains in protective pigmentation and photochemical acclimation (Yudina et al., 2022). Together, these findings show that breeding for superior performance under electric lighting should not treat light spectrum as a fixed background variable. Instead, breeders should explicitly target genotype × spectrum combinations, selecting cultivars whose photoreceptor signaling, photosynthetic efficiency, and metabolic outputs are optimized for the specific red, blue, and far-red light environments used in commercial CEA systems.

#### 3.2.2 Water and nutrient use efficiency

Water and nutrient management are critical in hydroponic systems for the efficient conversion of resources into biomass for sustainable CEA operations. Common hydroponic systems including nutrient film technique and DWC, along with their proprietary variants, are used in lettuce production, while other systems such as aquaponics, aeroponics, Ebb and Flow, drip and wick systems, and drip irrigation are less common. Breeding programs should focus on cultivars adapted to these systems with improved WUE and NUE. Quantitative trait locus (QTL) analysis has demonstrated that nitrogen, phosphorus, and water use efficiency traits are at least partly genetically independent and amenable to breeding improvement, with lettuce germplasm exhibiting substantial variation in nitrogen recovery efficiency, phosphorus uptake under low-phosphorus conditions, and the ability to maintain yield and biomass under reduced irrigation and nutrient availability (Eriksen et al., 2016; Macias‐González et al., 2021; Kreutz et al., 2023), providing a foundation for QTL-guided selection of resource-use-efficient cultivars.

Hydroponic lettuce production typically uses about 20 liters of water per kilogram of fresh weight, compared with about 250 liters per kilogram in conventional field production, a 92% reduction attributable primarily to closed-loop recirculation and controlled evapotranspiration rather than plant genetics (Barbosa et al., 2015). Genetic improvement in WUE within CEA therefore targets the residual variation that engineering and system design cannot capture. In a recombinant inbred population derived from *L. sativa* × *L. serriola*, QTLs for WUE-related traits such as carbon isotope discrimination (δ¹³C), transpiration rate, stomatal conductance, and yield each accounted for about 4–23% of phenotypic variance under well-watered and drought conditions, with a major hotspot where δ¹³C, stomatal conductance, and transpiration: dry-weight ratio co-localized under water limitation (Damerum et al., 2021). Accordingly, a breeding target for WUE in CEA lettuce can be to select genotypes with δ¹³C values, transpiration dry-weight ratio, or stomatal conductance lower than those of current commercial cultivars, indicating higher WUE, while maintaining or increasing fresh-weight yield under both ample and reduced water supply.

For NUE, a practical breeding target for CEA-adapted cultivars is the ability to sustain marketable shoot fresh weight at nutrient solution EC values near the lower end of the optimal hydroponic range (≈1.2 dS m^-1^), thereby reducing fertilizer inputs and limiting salt accumulation in recirculating systems. Association mapping in 142 butterhead cultivars grown under organic field conditions identified numerous marker–trait associations for water and nitrate capture and their use efficiencies, with significant genetic variation in nitrate acquisition from different soil layers and in shoot N accumulation across environments (Kerbiriou et al., 2016). Based on these results, quantitative selection goals for NUE in lettuce can be framed as increasing nitrate capture and shoot N content per unit of available N, i.e., higher nitrate and water capture efficiency and higher N use per unit resource while maintaining stable yield across contrasting environments. An experimentally validated target is achieving maximum biomass at a solution nitrogen concentration of ≤100 mg/L with an optimal NH_4_^+^-N:NO_3_^-^-N ratio of approximately 20:80, at which the highest lettuce biomass and nutrient (N and P) recovery rate have been reported in hydroponic systems (Xu et al., 2025). Together, these findings highlight the importance of improving WUE and NUE as key priorities for CEA breeding. Several seed companies, including Rijk Zwaan, Enza Zaden, BASF, Nunhems, and Syngenta have developed some lettuce cultivars for hydroponic and CEA production systems (Nunhems, n.d.; Syngenta Vegetables, 2023; Enza Zaden, n.d.; Rijk Zwaan USA, n.d.).

### 3.3 Optimizing plant architecture

CEA systems, especially vertical farms and PFALs, impose spatial constraints that differ from those of open-field production. In these systems, plant morphology must be optimized not only for biomass production but also for efficient use of water, nutrients, light, and three-dimensional growing space. Consequently, breeding for CEA must prioritize compact, efficient plant architecture suited to high-density production.

A key breeding priority for indoor lettuce is the development of cultivars with compact and uniform growth habits. In multi-tier systems, where plants are cultivated on vertically stacked shelves, limited clearance between production layers constrains plant height and canopy spread and can increase interference with lighting fixtures and harvesting equipment (Bhattarai et al., 2025). These engineering constraints define a practical architectural ideotype for vertical farming: compact stature, upright leaf angle, a dense but not overly self-shading rosette, and uniform growth across the crop. Such traits improve the use of volumetric space, enable higher planting densities, and increase productivity per unit area, a critical determinant of profitability in high-cost CEA systems. Uniformity is equally important for automation because robotic or mechanized systems designed around expected canopy dimensions perform less effectively when plant size and structure vary widely within a population.

Ideotype modeling provides a useful framework for translating these production constraints into measurable breeding targets for CEA lettuce. Rather than selecting only for general compactness, breeders can define an optimal rosette ideotype using traits such as plant height, leaf angle, leaf curvature, canopy density, and volume-to-biomass relationships that determine how efficiently a genotype occupies limited three-dimensional growing space under multi-tier cultivation. Recent 3D phenotyping work in lettuce showed that rosette structure, leaf density, and plant height can be quantified from reconstructed plant models (Bloch et al., 2025), while recent genetic analyses have identified loci controlling major leaf and architectural traits (Chen et al., 2025). Collectively, these advances support a model-informed breeding strategy in which architectural traits are integrated into multi-trait selection indices, allowing compactness, light interception, harvestable biomass, and automation compatibility to be improved simultaneously rather than in isolation.

Once a target CEA ideotype has been defined, breeding for compact stature can be pursued through genes regulating growth hormones, leaf orientation, and stem elongation. Traits such as reduced internode length, modified leaf angle, and controlled leaf size contribute directly to a more space-efficient architecture. In lettuce, *LsNRL4* overexpression reduces leaf angle while enhancing photosynthetic efficiency (An et al., 2022), producing a phenotype well suited to CEA. Similarly, a genome-wide association study (GWAS) using a multi-parent advanced generation inter-cross (MAGIC) population identified key regulatory genes such as *LsTCP4*, whose expression is negatively correlated with leaf curvature (Chen et al., 2025), providing additional targets for architecture optimization. Furthermore, double mutations in *LsKIPK* and *LsATPase* reduce leaf size and angle by altering cell wall development, generating compact butterhead lettuce architecture ideal for dense planting systems (Xie et al., 2024). Importantly, these targets should be evaluated not only under standard greenhouse conditions but also within the shelf spacing, lighting arrangements, and automation requirements of the intended commercial production system. Integrating 3D phenotyping and ideotype modeling can therefore help breeders balance the need for compact architecture with adequate canopy openness to minimize self-shading and maintain light-use efficiency. In this way, breeding for lettuce plant architecture for CEA moves beyond simple size reduction toward the development of lettuce cultivars matching the biological and engineering constraints of vertical farming.

### 3.4 Enhancing quality and consumer value

The highly controlled nature of CEA allows breeders to place greater emphasis on crop quality than is often possible in field production, where breeding priorities are frequently dominated by broad adaptation and stress resilience. In CEA, stable production conditions create opportunities to improve nutritional quality, sensory attributes, and postharvest performance while maintaining the consistency needed for premium markets (Bhattarai et al., 2025). This shift is commercially important because the high initial investment and operational costs of CEA must be offset not only by yield, but also by product differentiation and market value. Recent evidence suggests that consumer acceptance and willingness to pay for CEA lettuce are not automatically higher than for field- or greenhouse-grown lettuce (Krasovskaia et al., 2026) and remain highly heterogeneous, with consumers valuing perceptible attributes such as taste and freshness more consistently than the production method itself (Seong et al., 2026). Accordingly, premium-quality breeding goals are most likely to be commercially effective when they target traits that are consistent, recognizable, and economically meaningful rather than relying on indoor production alone as a premium signal.

#### 3.4.1 Nutritional content and functional components

Because indoor vertical farming is most economically viable when producing high-value crops, breeding should focus on lettuce with greater nutritional and functional quality that can support market differentiation. Shorter transport distances and ease of access in stores after harvesting further help preserve freshness and quality in CEA lettuce, increasing the commercial relevance of these traits. Most quality-related metabolites are quantitatively inherited and are influenced by both genotype and environment (Razzaq et al., 2022), making them suitable targets for breeding under the stable conditions provided by CEA. Significant genetic variation exists in lettuce for vitamins, pigments, sugars, and other bioactive components. These include vitamin C, β-carotene, anthocyanins, chlorophylls, tocopherols, phylloquinone, glucose, fructose, and sucrose. For example, studies in baby leaf lettuce have shown that some cultivars accumulate comparatively high concentrations of sugars and vitamin C, whereas others are distinguished by higher β-carotene, anthocyanins, or chlorophyll contents (Simko, 2019). Likewise, substantial variation in phylloquinone, α- and γ-tocopherols, and ascorbic acid has been documented in hydroponically grown lettuce (Murray et al., 2023), demonstrating clear potential for breeding nutrient-dense cultivars for CEA systems.

Anthocyanins are of particular interest because they contribute simultaneously to visual appeal, antioxidant capacity, and perceived functional value. These secondary metabolites are synthesized via the phenylpropanoid biosynthesis pathway and are widely regarded as health-promoting compounds with antioxidant and anti-inflammatory activity (Mattioli et al., 2020). Red leaf lettuce therefore represents an attractive breeding target for CEA, especially in premium salad markets. However, red pigmentation under artificial lighting can be inconsistent because anthocyanin accumulation in lettuce is governed by multiple genes and strongly influenced by environmental cues such as light spectrum and intensity (Wada et al., 2022), which may reduce visual quality and consumer appeal if coloration is unstable. Breeding for stable anthocyanin accumulation under CEA-specific light environments is therefore more commercially relevant than simply selecting for red color under field conditions. More broadly, nutritional enhancement in CEA is likely to be economically feasible when paired with visible quality traits or clear market claims, but less so when the nutritional improvement is not readily recognized or valued by consumers at purchase.

#### 3.4.2 Organoleptic quality

Organoleptic quality, including flavor, texture, aroma, and visual appearance, is central to consumer satisfaction and repeat purchase. Because lettuce is often consumed raw, these traits strongly influence market success, particularly in premium fresh-market and fresh-cut segments. In CEA systems, where environmental conditions can be tightly controlled, breeding can more effectively target sensory traits that are often unstable under open-field conditions (Bhattarai et al., 2025).

Breeding for conventional traits such as biotic and abiotic stress resilience has sometimes come at the expense of sensory quality (Folta and Klee, 2016). In lettuce, substantial variation and associated QTLs have been identified for sugars, organic acids, sesquiterpene lactones, and other metabolites that contribute to sweetness, bitterness, and overall flavor perception (Beharav et al., 2020; Chadwick et al., 2024). Environmentally stable QTLs, such as a major fructose QTL on LG 9 and a low-bitterness marker on LG 5 (Chadwick et al., 2024), provide useful targets for breeding superior flavor under CEA conditions. Factors like environmental and agricultural practices play a significant role in shaping vegetable quality, especially in terms of color, flavor, aroma, and the presence of key metabolites (Zhang et al., 2022; Rouphael et al., 2012; Chowdhury et al., 2021; Rouphael et al., 2018). Maintaining a stable environment is key to identifying metabotypes, which is one of the most time-consuming stages of breeding (Razzaq et al., 2022; Egorova et al., 2021; Weng et al., 2012; Zhang et al., 2022), whereas CEA production helps in creating ideal conditions for selected genotypes to express their optimal phenotype (Zhang et al., 2024). The precise environmental control in CEA minimizes environmental variation, enabling reliable metabotype identification and G×E optimization to enhance flavor expression and improve the breeding of superior sensory traits. Breeding efforts can now prioritize restoring or developing novel flavor profiles suitable for diverse consumer preferences. The higher demand for lettuce types that combine characteristics of leaf and crisphead varieties, known as “crunchleaf” or “summercrisp” within the industry (Bhattarai et al., 2025), illustrate how texture can contribute to differentiated consumer value in this crop.

Leaf texture is another critical trait affecting both eating quality and shelf life. Consumers often prefer soft, palatable leaves, whereas thicker and firmer leaves may better tolerate handling and transport. This creates an important breeding trade-off between immediate sensory quality and downstream postharvest performance. Because CEA allows precise control of environmental factors, breeders can use these systems to better distinguish genetic effects on texture and identify genotypes that maintain acceptable eating quality while supporting commercial handling requirements. Thus, the greatest economic value is likely to come from breeding strategies that improve flavor and texture in ways consumers can detect, while preserving the durability required for retail distribution.

#### 3.4.3 Postharvest quality and shelf life

For CEA to remain economically competitive with field-grown lettuce, cultivars must offer superior visual appeal and extended shelf life. Although CEA reduces wind damage and soil contamination, machine or robotic harvesting and extensive handling during packaging of fresh-cut and “ready-to-eat” markets could introduce substantial postharvest challenges. Thus, breeding for improved postharvest quality is a key priority in CEA systems.

Postharvest quality in lettuce is primarily defined by the resistance to two distinct physiological defects, enzymatic discoloration and rapid tissue deterioration (Peng and Simko, 2023). Enzymatic discoloration, perceived as browning or pinking, is a wound-healing response where mechanical damage upregulates phenylalanine ammonia-lyase (PAL), leading to the oxidation of polyphenols by polyphenol oxidases (PPO) into melanins (Saltveit, 2015; Teng et al., 2019; Hamdan et al., 2022; Sae-leaw and Benjakul, 2019). Distinct from this, tissue deterioration involves the loss of cell wall integrity and firmness, often exacerbated by low oxygen conditions (<2%) typical of modified atmosphere packaging (MAP), which can trigger anaerobic fermentation and off-odors (Hayes et al., 2014; Peng et al., 2020). Breeding for these traits is facilitated by their strong genetic basis, with broad-sense heritability estimates reaching 0.90 for deterioration rate (Simko et al., 2018a) and 0.92 for discoloration (Peng et al., 2021). Significant progress has been made using linkage mapping and GWAS, which identified *qSL4*, a major QTL on chromosome 4 that explains up to 74% of the phenotypic variation for deterioration rate (Hayes et al., 2014; Simko et al., 2018a; Sthapit Kandel et al., 2020). Additionally, the locus *qDEV7* has been identified as a key regulator of developmental rate, allowing breeders to optimize growth cycles without compromising longevity (Sthapit Kandel et al., 2020). Marker-assisted selection targeting these loci offers a precise method for selecting germplasm with superior shelf life.

Resistance to browning is regulated by specific QTLs such as *qDis1.1*, *qDis6.1*, and *qDis9.1*, which co-locate with genes encoding PPO and other oxidative enzymes (Peng et al., 2024; Hunter et al., 2022). These traits are highly responsive to preharvest and postharvest environments. Water deficit during the production phase has been shown to reduce browning potential in some crops by lowering polyphenol oxidase activity (Luna et al., 2013). Consequently, an integrated approach combining genetic selection for these loci with optimized handling practices is essential for extending the marketable shelf life of CEA-grown lettuce (Peng and Simko, 2023).

### 3.5 Stressors and physiological disorders

CEA systems are designed to minimize the impact of external biotic and abiotic stresses common in field cultivation. However, the highly optimized conditions that promote plant growth can also favor the development of diseases and insect pests if not properly managed. A key disease risk in CEA arises from the monoculture production of a single crop species within enclosed, recirculating systems, creating conditions fundamentally different from field agriculture. Once introduced through contaminated seed, transplants, water sources, equipment, or workers, pathogen inoculum can persist and rapidly amplify within recirculating nutrient solutions, ventilation pathways, and facility surfaces. Unlike field systems, CEA environments lack seasonal interruptions and often have limited microbial competition to suppress pathogen populations.

This structural vulnerability highlights the importance of resistance durability in CEA breeding programs. Consequently, breeding efforts should prioritize the development of durable, stacked resistance through gene pyramiding and proactive race monitoring, alongside the identification of new resistance sources and tolerance to CEA-specific biotic and abiotic challenges. In addition, tolerance to abiotic stresses remains important, as extreme temperatures, disruptions in light regimes, inadequate air circulation, and exposure through vents or unintended openings can compromise plant performance, ultimately reducing crop yield and quality.

#### 3.5.1 Abiotic stress and physiological disorders

##### 3.5.1.1 Temperature (heat and cold) stress tolerance

While CEA systems minimize the unpredictability of external weather, fluctuations in environmental parameters, particularly temperature, especially in tropical climates, can challenge the maintenance of optimal conditions and resource efficiency. Genetic improvement for both heat and cold tolerance will enable the development of lettuce cultivars optimized for stable performance, resource efficiency, and year-round production in CEA in tropical and temperate regions. Breeding for heat tolerance reduces the energy required for temperature regulation, thereby improving the economic viability of CEA systems. Although lettuce can handle low temperatures well, extremes can reduce yield and delay plant growth. Therefore, cold tolerance is an important trait in temperate regions. Zhao et al. (2022) demonstrated that lettuce adapts to consecutive heat stress through physiological and morphological modifications, including increased antioxidant enzyme activity, changes in photosynthetic pigments, and altered leaf morphology, which collectively work together to help mitigate the negative effects of heat stress on plant growth. Because these responses are influenced by genetic factors as well as environmental conditions, breeders can develop heat-tolerant lettuce cultivars by evaluating plant performance under CEA conditions.

Cold tolerance is an important breeding priority to reduce heating requirements and maintain year-round production in traditional low-tech greenhouses, especially in temperate regions or during winter. Park et al. (2024) identified a wild-type allele *LsCBF7* in wild lettuce (*L. serriola*), present at a high frequency (90%), controlling improved freezing tolerance. *LsCBF7* was linked to early bolting, an undesirable trait in cultivated lettuce (Park et al., 2024). This association likely led to negative selection of *LsCBF7* during domestication despite its adaptive advantage under cold stress. Breeding efforts should focus on decoupling freezing tolerance from early bolting using marker-assisted or genomic approaches. Use of genome editing can help in precisely transferring the locus, leaving the linked early bolting region behind to breed for cold tolerance.

##### 3.5.1.2 Bolting resistance

In lettuce, the transition from the vegetative rosette stage to the reproductive stage is marked by bolting. Because lettuce is harvested for its leaf rosette, bolting is highly undesirable; it reduces leaf biomass, causes elongated cores and cracked heads, and can lead to complete yield loss. The onset of flowering also induces biochemical changes that increase latex accumulation in leaves, resulting in bitterness and reduced market quality (Simonne et al., 2002). Therefore, late bolting is a key breeding target to extend the vegetative phase and maintain quality, particularly under high temperatures common in many production regions or under sub-optimal CEA systems.

Genetic variation for heat-induced bolting has been documented in leaf and crisphead lettuce types (Lafta et al., 2021, 2017; Lafta and Mou, 2013; Yoong et al., 2016; Jenni et al., 2013). Bolting is regulated by multiple interacting genetic pathways, including gibberellin (GA) signaling, *MADS*-*box* transcription factors, and temperature-responsive regulatory networks (Han et al., 2016). Key genes such as *LsKN1*, *LsRGL1*, *LsOFP6*, *LsFT*, and *BEL1*-like genes influence hormone signaling and stem elongation, while miRNAs and DNA methylation modulate environmental responses to heat and photoperiod, contributing to genotype-specific differences in bolting behavior (Han et al., 2016; Hao et al., 2022; Qi et al., 2025; Fukuda et al., 2017). A major QTL for bolting and flowering time, *qFLT7.2*, was identified on chromosome 7, and its effect is strongly influenced by photoperiod and temperature (Rosental et al., 2021).

##### 3.5.1.3 Tipburn resistance

Tipburn is a major physiological disorder in lettuce grown in CEA, causing inner-leaf margin necrosis that severely reduces marketability. Primarily driven by localized calcium deficiency due to poor transpiration in enclosed canopies (Barta and Tibbitts, 2000), tipburn fundamentally represents a supply-demand imbalance where rapid biomass accumulation outpaces calcium delivery (Simko and Ospina-Giraldo, 2026). CEA environments inherently aggravate this imbalance by maximizing growth rates through high light intensity, elevated temperatures, and high humidity (Collier and Tibbitts, 1984; Birlanga et al., 2021).

Breeding for tipburn resistance is highly feasible, supported by strong genetic variation across cultivated and wild *Lactuca* accessions (Beacham et al., 2023). Recent genetic studies have mapped major resistance QTLs on linkage groups 1 (*qTPB5.2*) and 5 (*qTBIN_1_QC11*), which frequently co-segregate with leaf morphology traits like crinkliness and head firmness (Macias-González et al., 2019, 2021; Jenni et al., 2013). Furthermore, genome-wide association studies have successfully decoupled resistance from plant vigor, identifying specific “cellular integrity” loci tied to calcium signaling and oxidative stress defense that confer resistance independently of rapid biomass accumulation (Simko and Ospina-Giraldo, 2026). Because tipburn exhibits strong genotype-by-environment interactions (Lafta et al., 2021), advancing CEA lettuce requires prioritizing production-system-specific screening (Beacham et al., 2023) to introgress these vigor-independent resistance alleles into high-yielding CEA cultivars.

##### 3.5.1.4 Salinity stress tolerance

Salinity presents a growing concern in certain production areas, especially where irrigation relies on saline aquifers or in coastal water sources. Lettuce is traditionally considered a salt-intolerant crop (Jácome Cavalcante et al., 2025). However, the development of cultivars tolerant to salinity is significant for CEA, particularly for their potential integration into non-traditional water management systems, such as saline water-based aquaponics systems. Genetic variability for salinity tolerance has been identified across different lettuce types, including crisphead, cos, and cutting types (Adhikari et al., 2019). However, the current levels of salt tolerance found are minimal (Adhikari et al., 2019). Breeding traits that maintain productivity under mild salinity could support CEA systems, particularly recirculating and aquaponic systems, by enabling efficient water and nutrient use in water-limited or urban environments.

Breeding efforts should target physiological traits that maintain productivity under mild salinity. Screening lettuce germplasm under limited water conditions helps identify resource-efficient genotypes, and improved salt tolerance would allow cultivars to perform well in recirculating and aquaponic systems where salts accumulate, enhancing water and fertilizer efficiency in urban or water-limited environments (Li et al., 2023).

#### 3.5.2 Biotic stress resistance

##### 3.5.2.1 Resistance to oomycete and fungal diseases

###### 3.5.2.1.1 Downy mildew

Downy mildew (DM), caused by the oomycete *Bremia lactucae*, is considered the most serious disease in lettuce production worldwide, and its significance in CEA is amplified by the humid, enclosed conditions that favor sporulation and by the continuous cropping cycles that sustain pathogen populations year-round. Rapid pathogen race evolution continuously requires the breeding community to identify new resistance sources and genes (Crute, 1992; Lebeda et al., 2014). While DM race BI:10US is prevalent in California and Arizona, races BI:37EU, Bl:38EU, Bl:39EU, Bl: 40EU, and BI:41EU are significant in Europe (IBEB, 2026). New DM resistant genes have been identified in *L. serriola*, *L. saligna*, and *L. virosa* and introgressed using repeated backcrosses and embryo rescue (Michelmore et al., 2012). At least 51 resistance genes, NLR immune receptor gene clusters (RGCs), and genomic regions have been identified and deployed in lettuce for DM resistance (Parra et al., 2016), and 38 *Dm* genes have been mapped (Parra et al., 2021). The priority breeding strategy should be stacking multiple *Dm* genes with different recognition specificities into elite CEA-adapted backgrounds, using marker-assisted and genome editing approaches to combine loci without linkage drag, thereby broadening the resistance spectrum and slowing pathogen adaptation. Continued monitoring of race composition in commercial CEA facilities, which may differ from field race distributions, is essential to ensure that deployed gene stacks remain effective.

###### 3.5.2.1.2 Powdery mildew

Lettuce powdery mildew (PM), caused by *Golovinomyces bolayi*, is a major issue in CEA systems due to the warm and humid conditions that favor disease development. Facilities with poor air circulation and inadequate temperature regulation, particularly in warmer regions, are especially vulnerable. The first report of PM infestation in a CEA facility has been recently documented (Ayala et al., 2025). Previous studies have shown that PM severity is negatively correlated with leaf thickness, with no disease development until the 10-leaf stage (Schnathorst, 1959). Varying levels of resistance have also been identified in *L. saligna* and *L. virosa* (Lebeda and Mieslerová, 2011). Among modern cultivars, leafy and butterhead lettuce types generally exhibit higher tolerance, whereas crisphead types tend to be the most susceptible (Simko et al., 2014). A mapping study in an interspecific population derived from a cross between susceptible *L. sativa* cv. Salinas and the highly susceptible *L. serriola* accession UC96US23 identified four significant QTLs, *pm-1.1*, *pm-2.1*, *pm-2.2,* and *pm-7.1,* on chromosomes 1, 2 and 7 that explained 35-43% of the phenotypic variation in the population (Simko et al., 2014). Two alleles, each conferring resistance were contributed by each parent demonstrating that resistance could be contributed by susceptible individuals (Simko et al., 2014). Although the parents showed varying levels of PM symptoms, these findings suggest that resistant or tolerant germplasm remains a valuable resource for discovering novel resistance genes. Importantly, several cultivars reported to be resistant in the field have been shown to develop PM symptoms when grown under greenhouse conditions (OSU Extension Service - Extension Communications, 2024). This illustrates that resistance expression can be further complexed by environmental factors, and that screening must be conducted in CEA facilities using pathogen isolates sourced from those systems.

###### 3.5.2.1.3 Pythium wilt (*Pythium* spp.)

Pythium wilt, a soil-borne disease, has been reported in lettuce cultivated in CEA, particularly in hydroponic systems (McGehee et al., 2018). Various *Pythium* species pose a significant CEA-specific threat because recirculating nutrient solutions in hydroponic systems provide a continuous, facility-wide transmission pathway once contamination occurs, enabling rapid pathogen spread throughout the system. These pathogens can establish and proliferate in circulating nutrient solutions, causing root rot, damping-off, wilting, and ultimately plant death.

Currently, no known resistance sources have been identified against *P. uncilunatum* or other *Pythium* spp. of concern to the CEA lettuce industry. Further investigation into the survival of these pathogens from contaminated seeds in soilless media (e.g., rockwool or vermiculite) could improve understanding of potential outbreak risks. Field evaluations of head, romaine, and leaf lettuce identified the cultivars Copious, Patton, and 1024 as showing the least disease symptoms, whereas most romaine types were susceptible (Smith et al., 2023). However, systematic screening of diverse lettuce germplasm against *Pythium* isolates obtained specifically from CEA systems is an urgent research priority, as the species composition and virulence profiles present in CEA may differ from those studied under field conditions. Additional research is needed to identify resistant sources and the genetic basis of resistance to the *Pythium* complex in CEA systems. In the absence of host resistance, biological control offers a promising near-term management strategy. For example, inoculation with *Pseudomonas* sp. strain IALR1619 has shown improved lettuce yield in plants infected by *P. ultimum* and *P. dissotocum* (Amaradasa et al., 2024), and it can be potentially used as a management tool while resistance sources are being developed.

###### 3.5.2.1.4 Fusarium wilt (*Fusarium oxysporum* f. sp. *lactucae* [FOL])

Fusarium wilt primarily occurs in warm regions worldwide under field conditions. *Fusarium* consists of three races (races 1, 2, and 3), which are mainly prevalent under field conditions. Although it is a soil-borne pathogen, FOL4 has recently emerged in lettuce grown under protected cultivation in northern Europe, first identified in the Netherlands and later spreading to the UK and Ireland (Clarkson, 2021). Cooler temperatures in CEA and the absence of crop rotation in monoculture CEA operations can favor the establishment and spread of *FOL4*, making these systems vulnerable to the disease. Screening of 96 accessions identified sources of resistance to FOL4 that could support the development of CEA-adapted resistant cultivars (Clarkson, 2021).

##### 3.5.2.2 Insect pest and virus resistance

###### 3.5.2.2.1 Impatiens necrotic spot virus

INSV, an orthotospovirus transmitted by the western flower thrips (WFTs; *Frankliniella occidentalis*), causes economic losses in lettuce due to spotted wilt symptoms. In recent years, the disease has become more widespread and severe in dry regions such as California, with incidences reaching up to 80% in some field trials. WFTs are also problematic in CEA because of their broad host range and ability to transmit INSV in protected systems. Greenhouse screening methods based on WFTs and mechanical inoculations were developed to screen 89 lettuce germplasms, revealing cultivars Amazon, Ancora, Antigua, Commodore, Eruption, Iceburg, La Brillante, Merlot, Telluride, and Tinto to be resistant (Simko et al., 2018b). A recent genetic study identified an environmentally stable QTL on LG2 in cultivar Eruption using recombinant inbred lines (RILs), explaining up to 61% of the phenotypic variance with a heritability estimate of 0.89 (Nayak et al., 2025). These resistance sources and QTLs may be used by breeding programs to develop molecular markers and apply them in breeding for INSV-resistant cultivars.

###### 3.5.2.2.2 Lettuce aphid (*Nasonovia ribisnigri*)

Lettuce aphids are a major pest of concern globally. Breeding for resistance to *N. ribisnigri* has been successful and deployed in modern cultivars, with resistance conferred by genes such as *Nr* to biotype Nr:0 (Eenink and Dieleman, 1983). However, a new biotype, Nr:1, emerged in Europe in 2007 and in Salinas Valley in 2018–2019, overcoming resistance in commercially available resistant cultivars (Thabuis et al., 2014). A Nr:1 biotype resistant lettuce was identified in *L. serriola* 10G.913571 (Thabuis et al., 2014). Additionally, two accessions of *L. virosa*, CGN16272 and CGN13355, showed resistance to Nr:1 biotypes (Cid et al., 2012). Further research is underway to investigate the underlying genes conferring resistance and develop novel resistant cultivars.

A comprehensive summary of all CEA-relevant lettuce breeding targets, detailing their known associated genetic resistance sources, phenotyping strategies, breeding methods, and current research gaps, is presented in **Table 1**.

**Table 1:** Comprehensive summary of controlled environment agriculture-relevant lettuce breeding targets, genetic resistance sources of traits of importance, phenotyping strategies, breeding methods, and current research gaps.

Trait/Breeding Target	Examples of Associated Gene(s) and/or QTL(s)	Phenotyping Method(s)	Breeding Method(s)/Tool(s)	Relevance to CEA	Research Gap(s)
Accelerated Growth Rate & Yield	Not reported	High-throughput phenotyping (HTP) using time-series RGB imaging for projected canopy size (PCS)	Genome-wide association study (GWAS), genomic selection (GS)	Shortened crop cycles maximize annual harvests and reduce lighting/energy costs per crop	Growth is bounded by sink strength; needs balance between rapid shoot expansion and carbon/calcium partitioning
Shoot-to-Root Biomass Ratio	Not reported	Biomass quantification (fresh and dry root and shoot mass) in hydroponics; HTP/PCS as a proxy for shoot biomass	GWAS, GS	Hydroponics eliminate the need for extensive roots, allowing energy redirection to marketable shoots	Lack of quantified optimal shoot-to-root ratios for specific hydroponic setups, and a need to identify genetic markers that suppress excess root growth without compromising shoot vigor.
Light Use Efficiency (LUE)	Not reported	Biomass quantification (fresh and dry root and shoot mass) in hydroponics under low light intensity; HTP/PCS as a proxy for shoot biomass	GWAS/QTL mapping	Lighting drives 10–50% of operating costs; high LUE reduces photon requirements	Understanding varying genotype-by-spectrum responses and optimizing light recipes for distinct cultivars
Water Use Efficiency (WUE)	*q13C6.1, q13C8.2, q13C9.1, q13C8.1, q13C9.2* (Damerum et al., 2021)	Carbon isotope discrimination (δ^13^C), HTP/hyperspectral/multispectral imaging for water index (WI)	GWAS, MAS, multi-omics integration	Captures residual variation that physical closed-loop CEA system design cannot optimize	Need to validate whether field-derived WUE QTLs (like those for carbon isotope discrimination) translate effectively to closed-loop, high-humidity CEA environments.
Nutrient Use Efficiency (NUE)	*LsaPHR1* (Qian et al., 2025), Lsat_1_v8_lg_5_224453011, Lsat_1_v8_lg_6_165637104 (phosphorus use efficiency; Kreutz et al., 2022), LSM00730 (nitrogen use efficiency; marker; Kerbiriou et al., 2016)	Biomass quantification under low nutrient conditions, HTP/multispectral red-edge chlorophyll index (RECI)	GWAS, MAS	Sustaining yields at lower nutrient concentrations limits fertilizer costs and hydroponic salt accumulation	Identifying genotypes that perform under specific hydroponic fertigation ratios
Compact Plant Architecture	*LsNRL4*, *LsTCP4*, *LsKIPK*, *LsATPase* (Chen et al., 2025)	HTP using 3D morphometric imaging (SFM-MVS) for volume-to-biomass modeling	GWAS, GS	High-density multi-tier planting requires uniform, space-efficient statures compatible with automation	Balancing compact stature with open canopies to prevent self-shading; validating platform-specific ideotypes
Nutritional & Functional Quality	*RLL4* (anthocyanin), *GGP1*, *GGP2* (β-carotene and ascorbic acid), *LCY-ϵ* (β-carotene ), *CCD4a* (β-carotene) (Livneh et al., 2025).	HTP using hyperspectral/multispectral imaging (e.g., NDAI for anthocyanin), untargeted/targeted metabolomic profiling	Genome editing/MAS, multi-omics integration	Premium traits (vitamins, anthocyanins) differentiate CEA products to offset operational costs	Anthocyanin accumulation can be unstable under artificial lighting; needs stable coloration targets
Organoleptic Quality	Fructose QTL on LG 9, low-bitterness marker on LG 5 (Chadwick et al., 2024)	Targeted Metabolomic profiling (e.g., sugars and sesquiterpene lactones for flavor traits)	GWAS, MAS, multi-omics integration	Flavor (sweetness, lacking bitterness) and texture (crunch) drive consumer satisfaction	Trade-off between desirable soft leaf texture and physical durability required for postharvest handling
Postharvest Quality	*qSL4*, *qDEV7* (Sthapit Kandel et al., 2020), *qDis1.1*, *qDis6.1*, *qDis9.1* (Peng et al., 2024)	Weekly visual score (1-10) of postharvest deterioration under modified atmosphere packaging, metabolomic/proteomic profiling (melanin, PAL, PPO)	GWAS, MAS, multi-omics integration	Essential for premium fresh-cut markets where browning and decay dictate shelf life	Integrating genetic selection with optimized handling practices to extend marketability
Temperature Stress Tolerance	*LsCBF7* (cold; Park et al., 2024), *LsNCED4* (heat; Bertier et al., 2018)	Targeted proteomic profiling of key relevant pathways (e.g., *MAPK* signaling and starch metabolism)	Genome editing, MAS, multi-omics integration	Optimizes performance across regions and reduces intensive HVAC heating/cooling requirements	Decoupling the wild-type *LsCBF7* freezing tolerance allele from premature bolting traits without linkage drag
Bolting Resistance	*LsKN1*, *LsOFP6* (Qi et al., 2025), *LsRGL1* (Wang et al., 2022), *LsFT* (Fukuda et al., 2017), *qFLT7.2* (Rosental et al., 2021), *SOC1* (Beracochea et al., 2023)	Visual screening and/or RGB-based monitoring for days to bolting and bolting severity	Genome editing, MAS	Prevents core elongation, head cracking, and bitter latex accumulation during environmental fluctuations	Decoupling resistance from rapid biomass vigor under optimal CEA conditions
Tipburn Resistance	*qTPB5.2* (Jenni et al., 2013), *qTBIN_1_QC11* (Macias-Gonzalez et al., 2019)	Visual and/or RGB-based screening for tipburn incidence and severity	GWAS, MAS	High growth rates under humid CEA conditions trigger severe localized calcium deficiencies	Introgressing vigor-independent "cellular integrity" loci so rapid growth does not compromise canopy integrity
Salinity Stress Tolerance	*qRC9.1, qRS2.1, qLS7.2* (Wei et al., 2014); *qSTgs2.1, qSTgs4.1, qSTgs4.2, qSTgs4.3, qSTgs7.1* (Das et al., 2024)	Visual stress rating; stress metabolite profiling (e.g., proline, MDA, antioxidants) and tissue ion concentration analysis (Na^+^, K^+^, Ca^2+^, Cl^-^) under saline irrigation	GWAS, MAS/GS, multi-omics integration	Enhances water/fertilizer efficiency in recirculating loops or saline water-based hydroponics/aquaponics	Need to identify novel, robust sources of salinity tolerance in diverse germplasm and wild *Lactuca* relatives, as current commercial germplasm exhibits only minimal tolerance
Downy Mildew Resistance	51 resistance genes/*NLR RGCs* (Parra et al., 2016), 38 *Dm* genes mapped (Parra et al., 2021)	Controlled inoculation and continuous RGB/Visual screening using facility-specific *Bremia lactucae* isolates.	Marker-assisted gene pyramiding (GS/MS)	Humid indoor monocultures and continuous cropping heavily favor year-round *B. lactucae* sporulation	Continuous monitoring of CEA-specific race evolution; combining loci without linkage drag to avoid resistance breakdown
Powdery Mildew Resistance	*pm-1.1*, *pm-2.1*, *pm-2.2*, *pm-7.1* (Simko et al., 2014)	Controlled inoculation and continuous RGB/Visual screening with CEA-specific isolates at/after the 10-leaf stage	GWAS, MAS	Warm indoor conditions with poor air circulation leave facilities vulnerable to outbreaks	Field-resistant cultivars often develop symptoms indoors; requires direct screening against CEA isolates
Pythium Wilt Resistance	Not reported	Controlled inoculation (root-dip assay) and screening against CEA-specific *Pythium* isolates.	GWAS/QTL mapping, MAS/GS	Soil-borne *Pythium* spp. amplify and transmit rapidly facility-wide via recirculating hydroponic solutions	No known host resistance identified; pathogen survival in soilless media is poorly understood
Fusarium Wilt Resistance (*FOL4*)	Not reported for FOL race 4; *qFOL7.1* and *qFOL8.1* reported for *FOL* race 1 (Seki et al., 2020); *qFOL1.2* for FOL race 2	Controlled inoculation (root-dip assay) and screening against *F. oxysporum* f. sp. *lactucae* Race 4 (FOL4) or other relevant isolates for specific CEA production systems, as needed.	GWAS/QTL mapping, trait integration through backcrossing and MAS	Cooler indoor temperatures and lack of crop rotation favor establishment of *F. oxysporum* f. sp. *lactucae*	Adapting known field resistance sources into CEA-adapted cultivars; identifying resistance sources for *FOL4*
Impatiens Necrotic Spot Virus (INSV)	*qINSV2.1* (Nayak et al., 2025)	Mechanical and/or thrips-mediated inoculation followed by greenhouse-based screening	GWAS/QTL mapping, MAS	Western flower thrips broadly transmit INSV within enclosed, protected systems	Applying newly discovered molecular markers in active breeding programs
Lettuce Aphid Resistance	*Nr* gene (Eenink and Dieleman, 1983) (reported for Nr:0 biotype but not for Nr:1 biotype)	Resistance screening against aphid biotypes (Nr:0, Nr:1) relevant to CEA	GWAS, Introgression from wild relatives (*L. serriola*, *L. virosa*) through backcrossing and MAS	Major pest globally; closed environments foster pest populations absent of natural predators	New biotype (Nr:1) overcomes standard resistance, requiring discovery and deployment of novel resistant genes

## 4 Breeding methods

Genetic improvement of lettuce for CEA can build on conventional selection and crossing while increasingly integrating molecular and computational tools to accelerate genetic gain and incorporate traits suited to indoor production systems, such as compact architecture, rapid but efficient biomass accumulation, RUE, and resistance to CEA-specific biotic and abiotic stresses. Advances in high-throughput phenotyping (HTP), genomics, and multi-omics now allow breeders to characterize complex traits with greater precision and to combine phenotypic and genetic marker data in predictive breeding frameworks. More recently, artificial intelligence and machine learning have expanded the capacity to process large image, sensor, and genomics datasets for trait extraction, genomic prediction, and genotype-by-environment modeling. However, the practical adoption of these approaches in lettuce breeding remains constrained by high computational costs, the need for large and well-annotated training datasets, limited cross-platform standardization, and still relatively sparse validation across breeding populations and commercial programs. For this reason, AI-based tools should currently be viewed as complements to, rather than replacements for, conventional breeding, phenotyping, and statistical genetics workflows.

**Figure 2:**
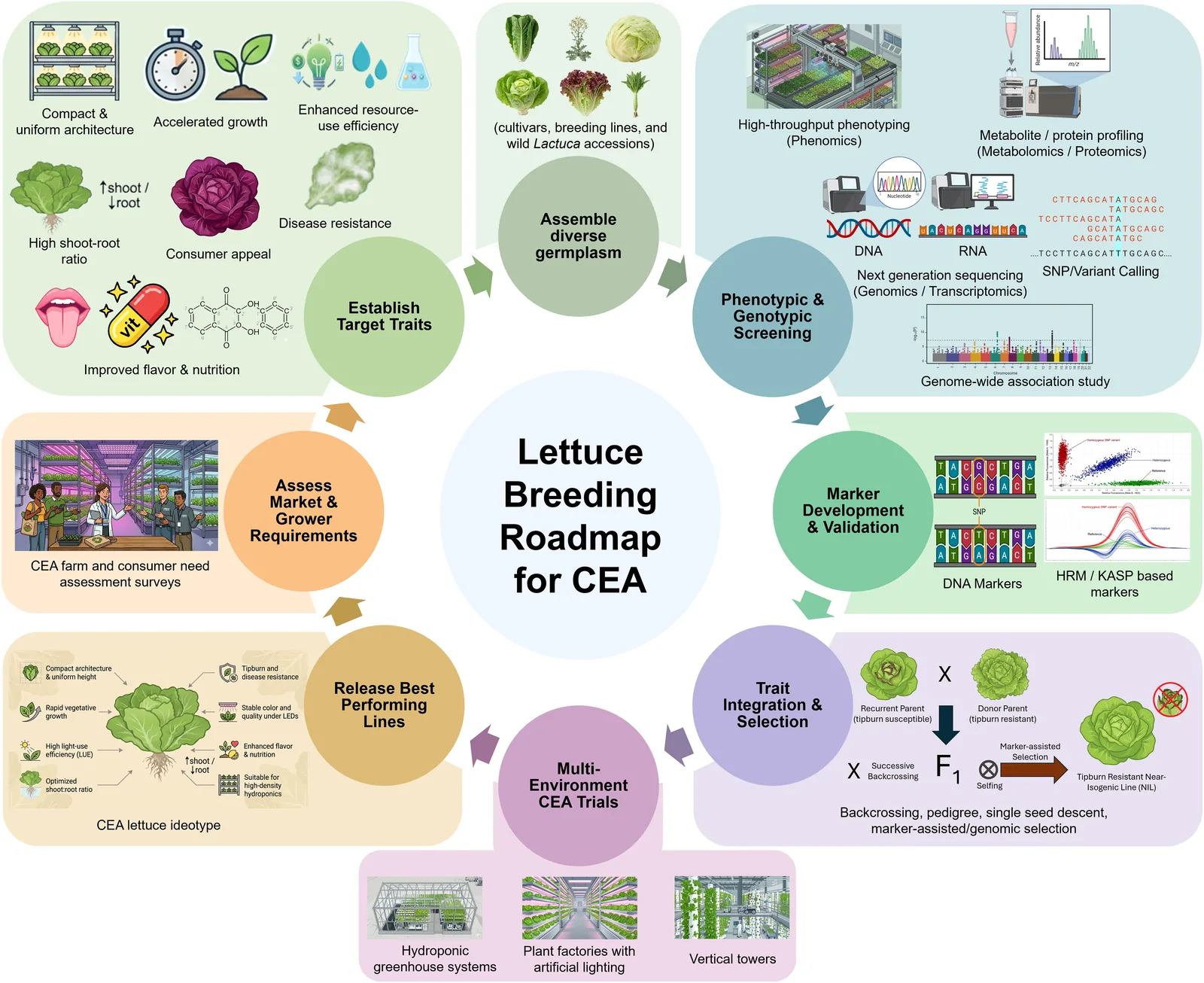
A roadmap to breeding lettuce for controlled environment production systems.

Modern breeding efforts therefore rely on advancements in multi-omics technology and HTP to accelerate the development of lettuce cultivars specifically tailored for CEA systems (Bhattarai et al., 2025; Kamran et al., 2023). These techniques enhance the precision, uniformity, and efficiency of selecting desirable traits such as high yield, stress resilience, and improved NUE, which are critical for the economic viability of indoor production. Genomics-informed breeding utilizes DNA markers to enable rapid and accurate selection of desired traits, effectively increasing the efficiency of breeding programs (Bhattarai et al., 2025). An overarching roadmap detailing the step-by-step integration of these modern screening technologies and classical selection methods to develop CEA-optimized lettuce cultivars is illustrated in **Figure 2**.

### 4.1 Traditional breeding techniques

Lettuce is a naturally self-pollinating diploid crop, which makes it well suited to conventional breeding approaches based on crossing, inbreeding, and selection over successive generations to develop stable, uniform lines. Consequently, most commercial cultivars available on the market are typically inbred lines (Sandoya, 2019). Core breeding methods include pedigree selection, single seed descent (SSD), and backcrossing. Historically, lettuce improvement relied heavily on pure line selection, followed by pedigree breeding in which individual plants are selected in segregating generations and advanced through selfing until desirable combinations of traits are fixed. Traits with high heritability are often selected in early generations (F_2_ to F_4_), whereas more complex traits that are strongly influenced by the environment and have low heritability are typically selected in later generations (after F_4_) (Sandoya, 2019).

**Figure 3:**
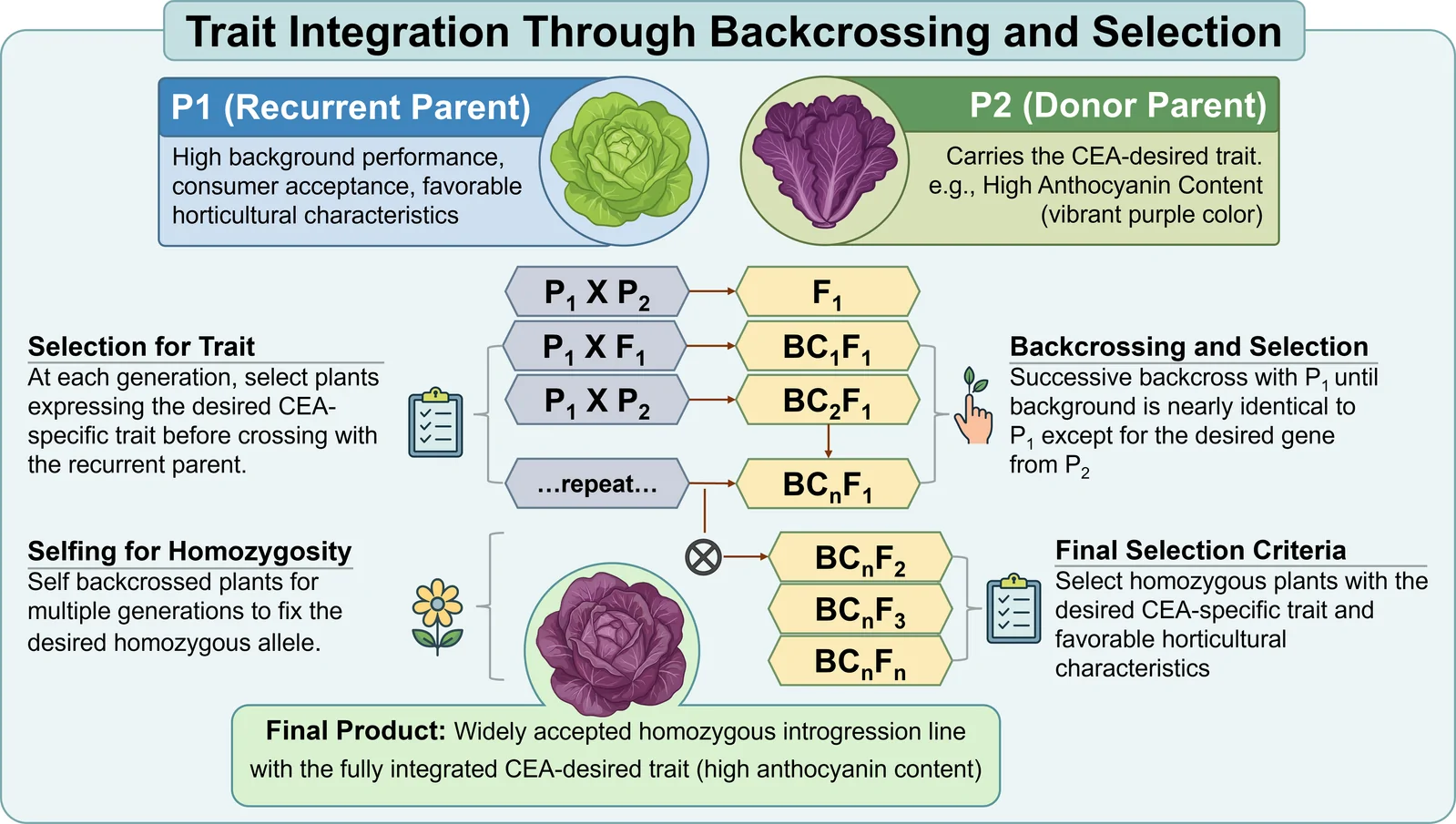
A conventional breeding approach for developing new lettuce cultivars for controlled environment agriculture.

Backcrossing remains especially useful for introgressing simply inherited traits controlled by a single gene or a few genes into elite germplasm. This technique involves crossing a desired donor parent with an elite recurrent parent lacking the specific characteristic, followed by repeatedly crossing the selected progeny back to the recurrent parent to restore the elite genetic background while maintaining the desired trait. In lettuce, this approach has been effective for disease resistance traits such as Verticillium wilt race 1 resistance, where the target allele can be recovered efficiently while restoring the recurrent parent background (Hayes, 2018). **Figure 3** illustrates an example of the stepwise process of introgressing a CEA-desired trait from a donor parent into an elite recurrent parent through backcrossing and selection. These conventional breeding strategies, despite being time-consuming and sometimes resulting in unfavorable traits due to linkage drag, are fundamental procedures that produce stable cultivars suitable for the predictable, uniform growing conditions of CEA (Hassan et al., 2021; Bhattarai et al., 2025). Despite the emergence of advanced molecular tools, classical breeding methods are indispensable for integrating complex, desirable traits such as compact growth, heat tolerance, disease resistance, and RUE into new lettuce cultivars (Hassan et al., 2021). RILs are frequently used to conduct mapping and genetic studies and for the improvement of complex traits (Beacham et al., 2023; Chadwick et al., 2024; Nayak et al., 2025; Peng and Simko, 2023; Selvakumar et al., 2025; Simko et al., 2014, 2025).

In lettuce, genetic diversity is generally limited due to its self-pollinating nature and long history of domestication. Consequently, accessions conserved in gene banks, including wild and primitive relatives, serve as vital sources of genetic variation. Interspecific hybridization is often used to introgress desirable traits from wild germplasm into cultivated lettuce. Three main wild species, *L. serriola*, *L. saligna*, and *L. virosa*, are known to harbor valuable traits, particularly related to biotic and abiotic stresses. Cross-compatibility between *L. sativa* and *L. serriola* facilitates the exchange of genomic regions between these species (Koopman et al., 1998). *L. serriola* is considered the wild progenitor of cultivated lettuce, and reciprocal crosses between the two result in fertile hybrids. Cross compatibility between *L. saligna* and *L. sativa* depends on the choice of accessions rather than unilateral incompatibility; however, the fertility of developed hybrids can be limited (de Vries, 1990). Notably, a cross between *L. sativa* and *L. virosa* produced the iceberg cultivar ‘Vanguard,’ which contributed to the development of modern iceberg types (Mikel, 2013). Additionally, primitive accessions such as oil-seed lettuce, although classified as *L. sativa*, retain wild-type characteristics and represent another important source of useful genetic variation. While limited hybrid fertility hinders traditional breeding efforts, techniques such as embryo rescue, somatic hybridization, genetic engineering, and genome editing may help overcome the barriers and facilitate the incorporation of valuable genes from less compatible species into modern cultivars (Hassan et al., 2021).

### 4.2 Marker-assisted selection

MAS employs DNA markers, such as single nucleotide polymorphisms (SNPs), that are tightly linked to genes controlling traits of interest. Markers are routinely used in lettuce breeding, particularly in private breeding programs as compared to public programs for cultivar fingerprinting, MAS, and genetic studies. The use of MAS in lettuce breeding has mainly focused on simply inherited traits controlled by a single gene or a few loci (Sandoya, 2019). Major applications include in selecting for disease resistance, biochemical compounds and pigmentation traits (Pink et al., 2022; Simko, 2013, 2023; Medina-Lozano et al., 2024; Kwon et al., 2013). Molecular markers on lettuce disease resistance has been previously described (Simko, 2013). Recently, advances in lettuce genomics and SNP arrays have enabled high-density genotyping for genetic studies, including diversity analysis, GWAS, QTL discovery, cultivar fingerprinting across broad germplasm such as collection centers or specific germplasm in a particular breeding program. Several SNP panels have been developed offering varying levels of saturation using different numbers of SNPs. A 6.6M GeneChipTM Array was developed by the Lettuce SFP Chip Project and made commercially available by ThermoFisher (ThermoFisher Scientific, n.d.; The Lettuce SFP Chip Project, 2016). This array detects single-feature polymorphisms in more than 35,000 genes covering whole genome and facilitates whole-genome QTL mapping, germplasm characterization, high resolution genome scans and high-density parallel genotyping. Similarly, SGS Institut Fresenius GmbH TraitGenetics Section developed 40K Axiom and 9K Infinium arrays that has been used in GWAS and genetic diversity studies (Medina-Lozano et al., 2024). These resources can be used in discovering new markers and genes that confer traits critical for CEA production.

### 4.3 Genomic selection

While MAS is highly effective for simply inherited traits controlled by a few major loci, genomic selection (GS) is designed for complex traits governed by many small-effect loci. GS uses genome-wide marker data to predict genomic breeding values, thereby enabling selection for complex traits even before phenotyping. In lettuce, the increasing availability of reference genomes, pan-genomic resources, and large genotyped populations has improved the understanding of genetic architecture and has created a stronger foundation for genomic prediction (Guo et al., 2023; Tripodi et al., 2023; Huo et al., 2026). In addition, multi-omics databases and technologies such as SPET genotyping provide large training datasets and data-generation capabilities for genomic prediction and GS studies (Guo et al., 2023; Tripodi et al., 2023). Several GWAS-based population studies coupled with high-density markers have demonstrated predictive potential in lettuce, laying the foundation for the application of genomic prediction (Park et al., 2021). Similarly, the precise quantification of highly heritable traits and the establishment of SNP-trait association further support the application of GS. MAGIC populations are particularly useful for training GS models because they offer high recombination, diverse haplotypes, and strong predictive capacity. The first MAGIC population, developed from 16 founder lines and 381 inbred progeny, with high genomic resolution and minimal population structure, enabled high-resolution analysis and the dissection of complex agricultural traits and leaf quality (Chen et al., 2025). These populations serve as ideal candidates for training GS models. GS-compatible models have been successfully applied, identifying 13 QTLs for leaf traits, enabling predictive breeding of leaf shape and anthocyanin content, which are critically important for CEA (Huo et al., 2026).

### 4.4 Artificial intelligence-mediated high-throughput phenotyping

High-throughput phenotyping has emerged as a transformative platform for lettuce improvement in CEA because it enables non-destructive, precise, and repeated measurement of complex morphological and physiological traits across developmental stages (Bhattarai et al., 2025; Tripodi et al., 2022). Lettuce is particularly well suited for HTP due to its compact canopy architecture and short life cycle, which facilitate rapid phenomic screening in greenhouses, growth chambers, and indoor vertical farms. Imaging-based HTP systems in CEA can be implemented using overhead camera platforms in greenhouse settings or conveyer-based imaging systems in vertical farms. These platforms can mount RGB, multispectral, hyperspectral, thermal, and 3D depth or LiDAR sensors to capture and record repeated, time-series phenotypic datasets across plant development. Such data provide not only precise morphological and physiological trait measurements but also enable detection of tipburn severity, nutrient deficiencies, early disease symptoms, and responses to abiotic stress. For accelerated breeding of CEA-adapted cultivars, non-invasive HTP methods are particularly valuable for early-stage trait prediction. For example, projected canopy size (PCS), measured early in development (8–13 days after seeding), is strongly correlated with final biomass and can be used for rapid screening (Jayalath, 2023). The integration of artificial intelligence (AI) further enhances the capacity and scalability of HTP systems (Sheikh et al., 2024). AI-driven approaches are increasingly used to automate trait extraction, improve prediction models, and enable data-driven breeding decisions by linking phenotypic, environmental, and genomic information. Modern CEA facilities equipped with imaging systems and environmental sensors can continuously capture high-resolution, plant-level data across developmental stages (Kaya, 2025). Deep learning architectures, including convolutional neural networks (CNNs) and transformer-based models, enable automated extraction of traits such as leaf area, canopy compactness, color uniformity, anthocyanin content, stress signatures, and growth dynamics (Han and Wang, 2025). Compared with manual scoring, these AI-enabled pipelines substantially improve throughput and consistency. Importantly, the integration of phenotypic and environmental data supports robust modeling of genotype × environment (G×E) interactions under controlled conditions, facilitating yield and quality optimization (Rahmonova et al., 2026). These models can also be used to simulate environmental scenarios, enabling selection of genotypes best suited for specific CEA systems. In addition, AI-based “digital twin” frameworks have been developed to simulate genotype performance over time under defined production environments, supporting targeted cultivar development (Liang et al., 2025).

Beyond phenotyping, AI is increasingly applied in genomic analyses. In sequencing workflows, deep learning improves variant calling accuracy by distinguishing true polymorphisms from sequencing noise (Abdelwahab et al., 2023). For genomic prediction, machine learning methods such as Random Forest, Gradient Boosting, Deep Neural Networks, and Bayesian deep learning often outperform traditional approaches like GBLUP and ridge regression by capturing non-linear marker–trait relationships and epistatic effects (Montesinos-López et al., 2025). AI-based approaches also enhance association mapping and gene discovery by detecting small-effect loci, modeling complex marker–trait relationships, and integrating multi-omics data beyond conventional QTL and GWAS frameworks (Mastropietro et al., 2026). Although the cultivated *L sativa* genome is well annotated, AI tools are particularly valuable for less-characterized wild *Lactuca* accessions, where they can assist in identifying sequence motifs, promoter regions, epigenetic patterns, and other regulatory elements. These models can further predict transcription factor binding sites, cis-regulatory elements, gene regulatory networks, and functional annotations of previously uncharacterized genes (Kim et al., 2021; Li et al., 2015; Wang et al., 2023). AI also has growing applications in genomics. When integrated with genome-wide marker data, AI-derived phenotypes further enhance genomic prediction accuracy, improve QTL resolution, and enable multi-trait selection indices tailored to CEA-relevant traits such as compact architecture, rapid biomass accumulation under LED lighting, and reduced physiological disorders. This integration accelerates the estimation of genomic breeding values and strengthens selection efficiency.

Nevertheless, several limitations continue to constrain the widespread adoption of AI in lettuce breeding. A major bottleneck is the requirement for large, high-quality, and well-annotated datasets spanning multiple environments, developmental stages, and genetic backgrounds, which remain limited and costly to generate. In addition, AI-based breeding pipelines often require substantial computational infrastructure, data storage, and specialized expertise (Sheikh et al., 2024; Han and Wang, 2025), limiting accessibility for smaller public breeding programs and some commercial operations. Limited standardization in imaging systems, lighting conditions, sensor calibration, data formats, and annotation protocols further reduces reproducibility and model transferability across platforms. Moreover, many AI models have been validated only under narrow experimental conditions and lack extensive testing across independent populations and commercial production environments. As a result, reported gains in prediction accuracy and breeding efficiency should be interpreted cautiously until broader validation and standardization are achieved.

Overall, the synergy between AI-enabled HTP and genomic tools provides a powerful framework for accelerating lettuce breeding. This integration shortens selection cycles, improves prediction accuracy, supports digital twin modeling of genotypes, and enables a systems-level understanding of trait architecture in controlled environments. Collectively, these advances facilitate the development of lettuce cultivars specifically adapted to CEA systems, improving productivity, consistency, and resource-use efficiency through data-driven breeding strategies.

### 4.5 Omics approaches

**Figure 4:**
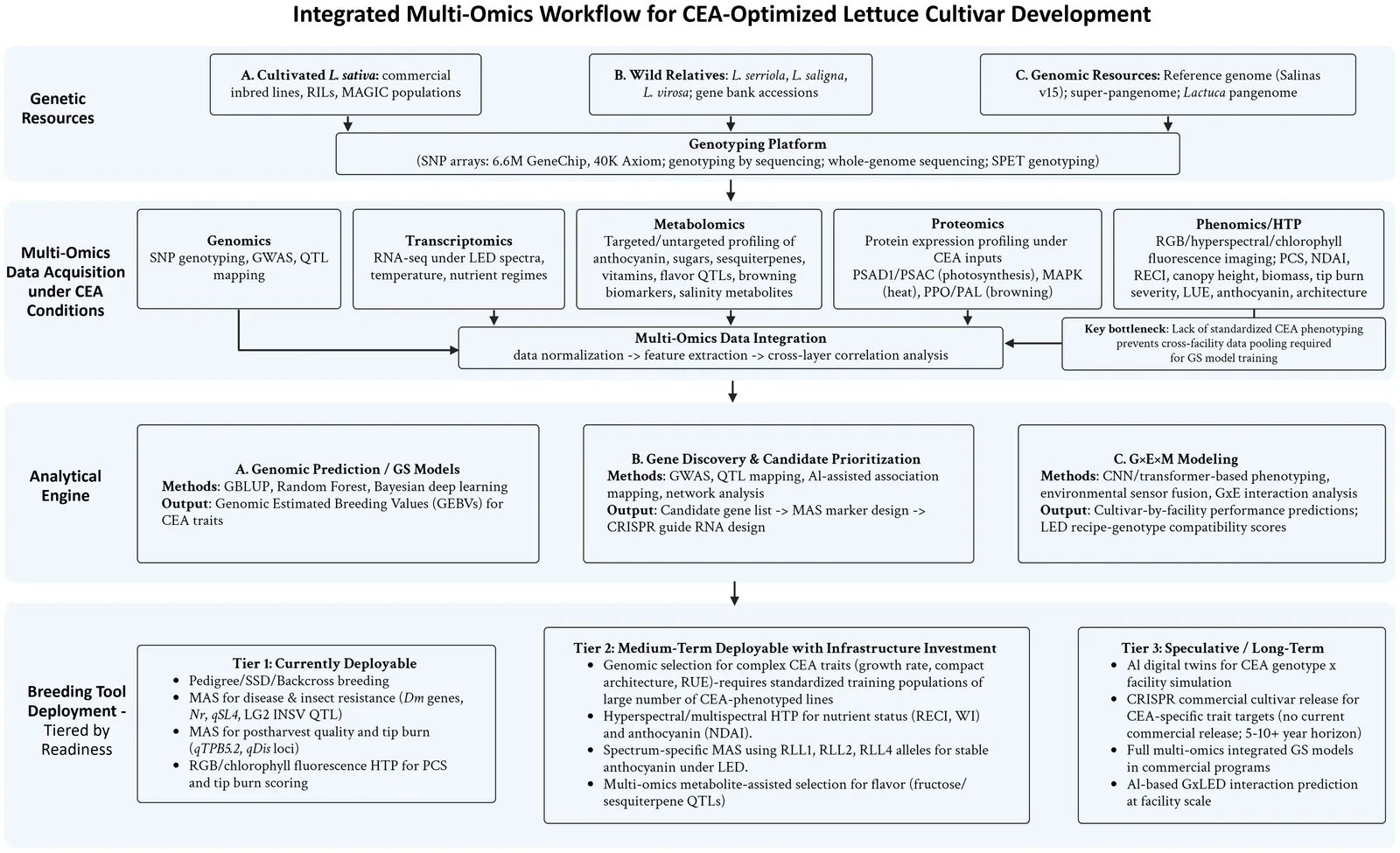
Integration of multi-omics approach in lettuce cultivar development specifically for controlled environment agriculture production systems.

The development and implementation of omics technologies have substantially expanded the breeder’s toolbox for crop improvement, driving a transition from traditional and marker-assisted breeding toward approaches that are increasingly precise, predictive, and data-driven. In lettuce, the integration of multiple omics platforms, including genomics, transcriptomics, proteomics, metabolomics, and phenomics, provides a comprehensive framework for understanding the molecular, biochemical, and physiological networks that connect genotype, gene regulation, and plant performance under controlled environment agriculture (CEA) conditions. Rather than functioning as isolated disciplines, these omics layers are most powerful when integrated, enabling the identification of genes, pathways, and biomarkers associated with key traits such as yield, morphology, nutritional quality, stress tolerance, and resource-use efficiency under artificial lighting, hydroponic systems, and tightly regulated climates. By linking genetic resources with CEA-specific phenotyping, data integration strategies, and advanced analytical pipelines, this multi-omics framework enhances the ability to predict and optimize lettuce performance in indoor production systems. As the demand for greater productivity and efficiency in CEA continues to increase, lettuce breeding will benefit from adopting this integrative omics-driven paradigm. An integrated multi-omics workflow for lettuce breeding in CEA is illustrated in **Figure 4**.

#### 4.5.1 Genomics and transcriptomics

The first lettuce reference genome was released in 2017 by sequencing cultivar Salinas (Reyes-Chin-Wo et al., 2017). An improved assembly of Salinas (Lsat_Salinas_v15) is available in the European Nucleotide Archive (ENA Browser, 2025). Additionally, improved genomes of cultivated lettuce including stem lettuce and genomes of wild relatives are available for *L. virosa*, *L. saligna*, and *L. serriola* (Cao et al., 2024; Shen et al., 2023; Wang et al., 2024; Xiong et al., 2023a, 2023). A super-pangenome of four *Lactuca* species comprising of 474 accessions is available (van Workum et al., 2024). Recently, a *Lactuca* pangenome has been developed by sequencing 10 accessions belonging to five cultivated morphotypes (butterhead, cutting, cos, Latin, and oilseed), one landrace, and four wild relatives (*L. serriola*, *L. saligna*, *L. virosa*, and *L. indica*) (Cao et al., 2025). These genomic resources will enable the discovery of new genes, loci, and SNPs, and large-scale variations including structural variations and copy number variations, while improving our understanding of lettuce evolution, domestication events, epigenetic features, and rare mutations for use in breeding lettuce with desirable characteristics. These genomic resources and genomics-associated technologies will minimize the time required for CEA breeding to achieve genetic gains compared with traditional approaches used for field breeding before these resources were available by precisely associating the genomic regions to the phenotypic traits of interest in CEA lettuce production.

Transcriptomics links this static genetic variation to environment-specific gene expression under CEA conditions. Transcriptomic profiling under different light–dark regimes has identified circadian hub genes such as *CIP1*, *SCL34*, and *ROPGEF1* that regulate growth, leaf morphology, soluble proteins, sugars, and carotenoids (Dai et al., 2024), providing regulatory targets for tuning biomass and nutritional profiles to CEA photoperiods. Light-response transcriptomics has revealed that *RLL4* integrates UV-B and blue-light signals via the *HY5–MBW* module, affecting flavonoid biosynthesis and color stability in red lettuce under artificial lighting (Yamashita et al., 2023). Other transcriptome studies have identified networks of *C2H2*, *AP2-EREBP*, and *WRKY* transcription factors mediating heat tolerance and bolting through gibberellin and auxin pathways; nitrate transporters (*NRT2.5*, *NRT1*) and antioxidant genes such as *LOX* responding to nitrogen limitation; and hormone biosynthesis genes induced by microalgal treatments or blue-light-enriched spectra that enhance growth and antioxidant capacity (Liu et al., 2018; Zhang Y, 2025; Santoro et al., 2023; Wang Y. et al., 2025; Zha et al., 2025; Liang et al., 2024). Together, these genomic and transcriptomic resources define specific loci, genes, and regulatory modules that can be targeted to develop CEA cultivars with improved growth, quality, stress resilience, and nutrient-use efficiency.

#### 4.5.2 Metabolomics and proteomics

Proteomics and metabolomics provide complementary functional readouts of how lettuce performs under CEA conditions. Proteomics captures changes in protein abundance and pathway activity, while metabolomics reflects the biochemical outcomes that directly shape nutritional quality, flavor, stress tolerance, and postharvest behavior. Together, these approaches help identify biomarkers linked to traits that can be difficult to infer reliably from genotype alone. For CEA breeding, their main value lies in connecting selection targets with measurable biochemical and physiological outcomes, such as metabolic signatures associated with nutritional value, bitterness, stress acclimation, or shelf life under hydroponic and indoor systems.

Metabolomics adds a biochemical layer that connects genotype and environment to traits directly valued in CEA markets, including nutritional quality, taste, color stability, and stress tolerance. Untargeted metabolite profiling across lettuce types and developmental stages has revealed substantial diversity and high heritability for specific metabolite classes, such as tannins in young plants and chalcones in mature leaves, indicating that these compounds can be efficiently improved through selection (Yang et al., 2018; Simko, 2024). Hydroponic production commonly alters the metabolome relative to soil-grown lettuce, increasing essential amino acids such as lysine, phenylalanine, and tryptophan (Hameed et al., 2022), and metabolomic signatures can be used to identify genotypes that consistently accumulate these health-promoting compounds under CEA fertigation and lighting regimes. Metabolite profiles also clarify the biochemical basis of flavor: sugars and terpenoids contribute to sweetness and aroma, while sesquiterpene lactones, including lactucopicrin, drive bitterness, particularly in stem lettuce (Chadwick et al., 2016; Wang et al., 2025; Zhang et al., 2026), highlighting pathway targets for tuning sensory properties using both cultivated and wild germplasm. In addition, specific metabolite ratios, such as ferulic acid methyl ester to caffeoylquinic acid, have been proposed as early biomarkers of postharvest browning, compounds such as adenosine and cis-aconitate reflect low-potassium stress, and broader secondary-metabolism reprogramming characterizes melatonin-associated salinity responses (García et al., 2018; Gao et al., 2022; Secomandi et al., 2025; Yang et al., 2024). These markers guide selection for genotypes that maintain visual quality and metabolic homeostasis in recirculating CEA systems.

Proteomics complements metabolomics by quantifying functional proteins that mediate these metabolic and physiological changes. Studies of lettuce fertilized with organic composts or fulvic acid have reported increased accumulation of photosystem components (*PSAD1*, *PSAC*) and other light-harvesting proteins, supporting carbon assimilation and biomass production even in the presence of heavy metals, and thereby identifying genotypes that respond favorably to organic inputs and biostimulants often used in sustainable hydroponics (Cappelini et al., 2025; Chen et al., 2022). Under heat stress, exogenous spermidine has been shown to upregulate proteins involved in *MAPK* signaling and carbohydrate metabolism, particularly starch and sucrose pathways, which was associated with a more stable energy supply and improved growth at elevated temperatures and suggests candidate protein markers for thermotolerance in CEA cultivars (Sun et al., 2022). Postharvest proteomic analyses have linked extended shelf life and reduced browning to changes in proteins such as protein disulfide isomerase and glutamine synthase as well as altered *LOX* and phenylalanine ammonia-lyase activities under modified atmosphere packaging (Weng et al., 2024; Zhang et al., 2023), pointing to protein-level targets that could be considered when breeding lettuce for improved texture and color retention during fresh-cut storage.

#### 4.5.3 Phenomics and multi-omics data integration

High-throughput phenotyping and phenomics provide a practical link between molecular variation and CEA performance traits by enabling rapid, non-destructive measurement of growth, architecture, stress responses, and quality under production conditions. Lettuce’s short production cycle and relatively simple canopy make it well suited for image-based phenotyping in indoor systems. Imaging approaches have been applied in greenhouses and vertical farms to repeatedly quantify canopy development, biomass proxies, pigment-related indices, and stress signatures across many plants and time points without destructive sampling (Bhattarai et al., 2025; Kim, 2022; Kaya, 2025; Li et al., 2025; Zhao et al., 2025a; Ojo et al., 2024; Tripodi et al., 2022). Early projected canopy size obtained from chlorophyll fluorescence imaging correlates strongly with final shoot dry weight in green lettuce cultivars (Kim and van Iersel, 2022), indicating that such metrics can be useful for identifying genotypes with desirable canopy structure, biomass accumulation, tipburn response, and resource-use characteristics in CEA (Kim, 2022; Kim and van Iersel, 2022; Ojo et al., 2024). Image analysis and associated machine-learning methods can further refine trait extraction, enabling precise quantification of traits such as plant height, crown width, leaf area, and fresh and dry mass from image data (Li et al., 2025; Zhao et al., 2025b; Ojo et al., 2024). Phenomics also helps reveal physiological trade-offs relevant for breeding. In red lettuce, elevated anthocyanin content has been associated with reduced light-use efficiency and weaker relationships between canopy size and biomass, likely because pigments intercept part of the incident light (Kim and van Iersel, 2022). To separate pigment and growth signals, indices such as the normalized difference anthocyanin index (NDAI) have been developed to quantify anthocyanin concentration, supporting efforts to balance visual or nutritional attributes with yield under particular LED lighting conditions (Kim, 2022). Beyond morphology and pigmentation, HTP has been used to study eco-physiological responses and resilience to abiotic stresses common in indoor production, including varying vapor pressure deficit (VPD), salinity, and limitations in water or nutrients. Hyperspectral-based vegetation indices, for example, the red-edge chlorophyll index (RECI) for nitrogen status and the water index (WI) for drought-related responses, can detect changes in plant status before severe symptoms develop and have been used to screen genotypes for performance under reduced inputs (Weksler et al., 2022). High-throughput seedling assays have identified differences in salt sensitivity that, when confirmed at later stages, help pinpoint germplasm with improved performance in recirculating hydroponic systems where salts may accumulate (Laing, 2025). Integrating image-based phenotyping with anatomical measurements, such as stomatal and vein density and mesophyll organization has provided insight into how lettuce varieties respond to rapid shifts in VPD and related microclimatic factors (Amitrano et al., 2022).

The main opportunity going forward is to integrate phenomics within a broader multi-omics breeding framework rather than expanding each data layer in isolation. In practice, a CEA-oriented multi-omics workflow begins with diverse germplasm and controlled-environment phenotyping, then combines genomic variation with transcriptomic, proteomic, and metabolomic profiles to identify candidate loci, pathways, and biomarkers before validating these in breeding populations under target CEA conditions. The transformative power of this approach lies in integrating genomic, transcriptomic, proteomic, metabolomic, and phenomic datasets into AI-based predictive models. Machine learning and AI approaches, including random forest, gradient boosting, deep neural networks, backpropagation neural networks, and Bayesian deep learning, are increasingly used to fuse multi-layered data into multi-trait prediction models that capture non-linear and epistatic effects and often outperform traditional GBLUP-based genomic selection (Montesinos-López et al., 2025; Guo et al., 2023; Tripodi et al., 2023; Li et al., 2025; Zhang X et al., 2025). These AI-driven models help elucidate complex genotype–phenotype–environment relationships, linking specific alleles and metabolic signatures directly to observable, highly heritable CEA traits (Chauhan and Singh, 2025). Within the integrated workflow shown in **Figure 4**, multi-omics prediction models can translate diverse data streams into genomic estimated breeding values for CEA-relevant traits, allowing breeders to prioritize crosses, shorten selection cycles, and design cultivars tailored to the economic and environmental constraints of specific lettuce production systems. Coupled with “speed breeding” in controlled environments that maintain continuous optimal growth, this framework can substantially compress breeding timelines and accelerate the development of next-generation lettuce ideotypes with tailored nutritional profiles, optimized architectural forms, and robust stress resilience for sustainable, high-density indoor lettuce production (Sharma et al., 2024).

### 4.6 Genome editing

Targeted modification of the lettuce genome through genome editing has enabled direct improvement of traits that traditionally required introgression from compatible or incompatible wild relatives, thereby helping overcome barriers imposed by interspecific crossing incompatibilities. Traits such as thermotolerance, present in wild relatives, have been introduced into cultivated lettuce by editing *LsNCED4* (Bertier et al., 2018), while undesirable traits such as premature bolting have been successfully reduced through genome editing (Choi et al., 2022). Nutritional compounds such as ascorbic acid, β-carotene, and lactucaxanthin have also been simultaneously enhanced by editing regulatory regions in the upstream ORF of *GDP-L-galactose phosphorylase 1* and *2* (u*GGP1* and u*GGP2*), *lycopene ϵ-cyclase* (*LCY-ϵ*), *carotenoid cleavage dioxygenase 4a* (*CCD4a*) in romaine lettuce (Livneh et al., 2025). A single base mutation achieved using CRISPR-Cas9 system in *SOC1* has successfully delayed first flower bud formation and expression of flowering regulation genes including *LsLFY*, *LsFUL*, *LsAPL1*, and *LsAPL2* and can be used to achieve higher yields through delayed bolting (Beracochea et al., 2023). CRISPR-mediated gene editing has further been applied to develop resistance to white mold (*Sclerotinia sclerotiorum*) (Dybdal, 2024). Traits such as high yield, delayed bolting, increased nutritional content, disease resistance, and thermotolerance are important in lettuce production in CEA for decreased production cost, optimized production, and attract price premiums. Therefore, gene editing will be an important tool in the breeders’ toolbox to optimize lettuce traits for CEA.

Practical deployment of CRISPR-Cas9, however, will depend not only on trait efficacy but also on regulatory approval, editing quality, and market acceptance. Regulatory treatment of gene-edited crops currently varies across jurisdictions, with several countries evaluating certain non-transgenic edits more like conventional breeding outcomes, whereas the European Union has generally maintained stricter oversight; this uneven landscape affects international deployment and trade of genome-edited lettuce (FDA, 2024; Venkataraman et al., 2025; Ricroch et al., 2026). According to a comparative study, public perceptions of gene-edited crops vary across countries. Consumers in the U.S. tend to perceive greater benefits and lower risks than those in Germany, while Japanese consumers hold intermediate views, exhibiting risk perceptions similar to those of German participants but benefit perceptions closer to those of U.S. participants (Kato-Nitta et al., 2023). At the technical level, a recent optimization of gene-editing system in lettuce has shown that the lettuce endogenous *LsU6–10* promoter drives CRISPR/Cas9 guide RNA expression much more efficiently than the traditionally used *AtU6–26* promoter from *Arabidopsis thaliana* (Riu et al., 2023). In addition, freedom to operate may be constrained by intellectual property related to CRISPR systems, editing reagents, and elite germplasm (Ricroch et al., 2026), which may slow public-sector deployment even when proof-of-concept edits are successful. At the same time, the possibility of unintended edits at off-target sites remains a key biosafety and regulatory concern, and current guidance emphasizes careful design, molecular characterization, and, where possible, whole-genome screening to detect unintended changes (Prakash et al., 2026). Commercialization of gene-edited lettuce remains limited globally, but romaine lettuce is now among the few crop species to have reached commercialization in the gene-edited plant pipeline, following regulatory clearance and commercial availability of the non-browning cultivar ‘GVR-108XL’ (GreenVenus, LLC, 2022). This indicates that lettuce is moving from proof-of-concept toward market deployment, even though edited lettuce products are not yet widely established across major markets.

## 5 Conclusion

The accelerating expansion of CEA presents a climate-resilient approach to meeting the growing global demand for fresh, high-quality lettuce. However, realizing the full productive and economic potential of these systems requires more than engineering innovations alone. The mismatch between cultivars developed for variable open-field conditions and the demands of high-input, space-limited, and tightly regulated indoor environments represents a critical bottleneck that genetic improvement is uniquely positioned to address. Closing this gap demands a paradigm shift in breeding priorities away from broad field adaptation and toward ideotypes explicitly optimized for the biological, operational, and economic realities of CEA. CEA eliminates many of the environmental stressors that have historically dominated lettuce breeding, yet it imposes a distinct set of challenges of its own. High capital and operating costs, particularly for lighting and climate control, make small gains in LUE, growth rate, HI, and RUE economically consequential, especially in vertical farms and plant factories that depend exclusively on electric lighting. Concurrently, the enclosed, recirculating nature of these systems amplifies vulnerability to persistent pathogen inoculum and physiological disorders such as tipburn, which arise when ultrafast growth outpaces calcium delivery and sink capacity. These interacting pressures argue for breeding strategies that treat CEA not just as a modified field environment, but as a fundamentally distinct agroecosystem with its own optimal trait combinations, performance thresholds, and trade-offs.

Across the evidence synthesized in this review, four interconnected breeding domains emerge as central to improving CEA lettuce: accelerated growth and improved HI; enhanced RUE; architecture suited to multi-tier systems and automated production; and stable, high product quality including postharvest performance. Within each domain, modern breeding tools, multi-omics integration, HTP, artificial intelligence, and gene editing collectively provide a powerful and rapidly expanding toolbox for dissecting complex traits, modeling genotype-by-environment-by-management interactions, and accelerating genetic gain, especially because CEA facilities themselves can function as speed-breeding platforms. Importantly, however, these tools must be deployed within a coherent breeding framework that explicitly links trait targets to economic and operational outcomes, rather than pursuing biological optimization in isolation from the commercial realities of indoor production.

Despite these promising opportunities, several significant limitations and practical barriers must also be considered. Biologically, breeding for ultra-fast growth and shortened crop cycles is constrained by physiological trade-offs: tipburn arises from source-sink imbalances and localized calcium deficiencies in rapidly expanding canopies, and lettuce yields under optimized light increasingly become increasingly sink-limited, meaning photosynthetic improvements must be matched by enhanced cellular capacity to store and partition assimilates. Technologically, the widespread adoption of AI and multi-omics prediction models is currently limited by high computational costs, lack of cross-platform standardization, and the requirement for large, well-annotated training datasets validated across diverse commercial environments. From a regulatory and market standpoint, the commercial deployment of gene-edited cultivars faces complex intellectual property constraints, variable global regulatory frameworks, and uncertain consumer acceptance, all of which can substantially delay the translation of laboratory advances into commercially ready cultivars. Finally, and perhaps most importantly, genetic improvement alone cannot overcome the fundamental capital and energy constraints of indoor farming, and biological optimization must therefore work synergistically with engineering innovations and economically sound production practices rather than being expected to compensate for their limitations. **Table 2** presents a proposed priority framework for lettuce breeding in CEA, outlining short-, medium-, and long-term objectives based on trait complexity and technological readiness. Taken together, these considerations define the following five priority directions for future research to support CEA lettuce breeding:

**Table 2:** Framework for lettuce breeding in CEA, outlining short-, medium-, and long-term objectives based on trait complexity and technological readiness.

Time Horizon	Priority Focus & Rationale	Key Breeding Targets	Primary Breeding Tools & Methods
Short-Term (1–3 Years)	Deployment of Known Loci: Focuses on traits with high heritability and established major QTLs or genes, offering immediate economic impact with minimal discovery lag.	Postharvest Quality: Extending shelf life and reducing enzymatic browning (*qSL4*, *qDEV7*, *qDis* loci). Targeted Biotic Resistance: Deploying known *Dm* genes for downy mildew and the *qINSV2.1* for INSV resistance.	MAS, gene pyramiding, and conventional backcrossing into elite CEA backgrounds.
Medium-Term (3–7 Years)	Resolving Trade-offs & Linkage Drag: Focuses on complex traits that require mitigating physiological trade-offs or utilizing advanced phenotyping to optimize the plant's physical and metabolic response to indoor environments.	Decoupled Tipburn Resistance: Introgressing vigor-independent alleles so rapid growth does not trigger calcium deficiencies. Compact Architecture: Targeting *LsNRL4*, *LsTCP4*, and related genes for automation-friendly, high-density multi-tier growth. Temperature Adaptation: Decoupling cold tolerance (*LsCBF7*) from early bolting to lower HVAC energy loads. Stable Nutritional Quality: Ensuring consistent anthocyanin and metabolite accumulation under artificial LEDs.	HTP, 3D imaging, CRISPR/Cas9 genome editing, and GS using MAGIC populations.
Long-Term (7+ Years)	Systemic Optimization & Novel Discovery: Focuses on highly quantitative traits and entirely new disease threats that require de novo discovery, complex modeling, and integration of the plant with facility engineering.	Resource Use Efficiency: Optimizing light (LUE), water (WUE), and nutrient (NUE) use efficiency to lower fundamental operating costs. Novel Disease Resistance: Identifying new resistance sources for soil-borne hydroponic pathogens like *Pythium* wilt. Holistic CEA Ideotypes: Simultaneous, multi-trait optimization customized to specific vertical farm or plant factory constraints.	AI-driven genomic prediction, digital twin modeling, multi-omics integration, and de novo screening of wild *Lactuca* germplasm.

1. Optimizing genotype-specific resource-use efficiency: Future research must systematically characterize genotype × LED spectrum interactions to develop cultivars with superior light-use efficiency at reduced energy inputs, while simultaneously improving nitrogen and water capture to sustain high yields under low-input hydroponic fertigation strategies. Identifying genotypes that maintain marketable quality under lower photon flux densities is especially urgent given that lighting accounts for the majority of operating costs in plant factories.
2. Designing and validating CEA-specific architectural ideotypes: Breeding programs should employ 3D phenotyping and AI-driven ideotype modeling to select for compact, uniform plant architectures with optimized shoot-to-root biomass partitioning, enabling higher multi-tier planting densities and compatibility with robotic harvesting systems. These efforts should be coupled with multi-environment trials across greenhouse, vertical farm, and plant factory platforms to determine the extent to which platform-specific ideotypes are necessary rather than broadly applicable.
3. Decoupling rapid growth from physiological disorders: A critical research priority is identifying and introgressing vigor-independent resistance alleles for tipburn and premature bolting, ensuring that ultra-fast biomass accumulation under optimal CEA conditions does not compromise canopy integrity or marketable yield. This will require detailed physiological and genetic dissection of calcium translocation dynamics, sink capacity, and source-sink regulation at high planting densities and light intensities.
4. Developing durable, CEA-specific disease resistance: Breeding programs must prioritize the pyramiding of multiple resistance genes against priority enclosed-system pathogens, including downy mildew, Pythium wilt, and powdery mildew, together with continuous, facility-specific monitoring of pathogen race evolution to prevent resistance breakdown in the genetically uniform canopies typical of commercial CEA operations.
5. Standardizing AI and multi-omics integration across platforms: The research community must establish open-access standardized HTP protocols and leverage emerging pan-genomic and super-pangenomic resources to build robust genomic prediction models and digital twin frameworks that accurately forecast genotype-by-environment interactions across diverse CEA configurations. Such infrastructure will reduce the data burden on individual breeding programs, improve the transferability of prediction models across platforms, and ultimately accelerate the selection cycles for complex, multi-trait CEA ideotypes.

Collectively, these priorities illustrate that breeding lettuce for CEA is not only a scientific priority, but also an economic necessity for the industry. By aligning genetic improvement with the operational and economic realities of controlled environments, addressing known biological trade-offs, and integrating breeding efforts with engineering advances and consumer insights, breeding programs can help transform lettuce into a crop that fully realizes the potential of CEA, supporting more resource-efficient, profitable, resilient, and quality-focused production systems capable of meeting the food demands of a rapidly growing and increasingly urbanized global population.
